# Adapalene and Doxorubicin Synergistically Promote Apoptosis of TNBC Cells by Hyperactivation of the ERK1/2 Pathway Through ROS Induction

**DOI:** 10.3389/fonc.2022.938052

**Published:** 2022-07-06

**Authors:** Umar Mehraj, Irfan Ahmad Mir, Mahboob ul Hussain, Mustfa Alkhanani, Nissar Ahmad Wani, Manzoor Ahmad Mir

**Affiliations:** ^1^ Department of Bioresources, School of Biological Sciences, University of Kashmir, Srinagar, India; ^2^ Department of Biotechnology, School of Biological Sciences, University of Kashmir, Srinagar, India; ^3^ Emergency Service Department, College of Applied Sciences, AlMaarefa University, Riyadh, Saudi Arabia; ^4^ Department of Biotechnology, School of Life Sciences, Central University of Kashmir, Ganderbal, India

**Keywords:** breast cancer, TNBC, doxorubicin, adapalene, Chou-Talalay, combination therapy, drug resistance

## Abstract

Doxorubicin is a commonly used chemotherapeutic agent to treat several malignancies, including aggressive tumors like triple-negative breast cancer. It has a limited therapeutic index owing to its extreme toxicity and the emergence of drug resistance. As a result, there is a pressing need to find innovative drugs that enhance the effectiveness of doxorubicin while minimizing its toxicity. The rationale of the present study is that combining emerging treatment agents or repurposed pharmaceuticals with doxorubicin might increase susceptibility to therapeutics and the subsequent establishment of improved pharmacological combinations for treating triple-negative breast cancer. Additionally, combined treatment will facilitate dosage reduction, reducing the toxicity associated with doxorubicin. Recently, the third-generation retinoid adapalene was reported as an effective anticancer agent in several malignancies. This study aimed to determine the anticancer activity of adapalene in TNBC cells and its effectiveness in combination with doxorubicin, and the mechanistic pathways in inhibiting tumorigenicity. Adapalene inhibits tumor cell growth and proliferation and acts synergistically with doxorubicin in inhibiting growth, colony formation, and migration of TNBC cells. Also, the combination of adapalene and doxorubicin enhanced the accumulation of reactive oxygen species triggering hyperphosphorylation of Erk1/2 and caspase-dependent apoptosis. Our results demonstrate that adapalene is a promising antitumor agent that may be used as a single agent or combined with present therapeutic regimens for TNBC treatment.

## Introduction

Breast cancer (BC) is the most frequent cancer in women, with an estimated 2.2 million cases diagnosed in 2020 (1). Presently, BC is the main reason for global tumor-related deaths (2). Triple negative breast cancer (TNBC) is a highly invasive and aggressive BC subtype, accounts for 15% to 20% of all BCs, and lacks hormonal receptors and HER2 amplification (3). TNBC patients tend to show poor prognosis owing to its aggressive nature and limited therapies (4, 5). Additionally, TNBC has a high proclivity for rapid recurrence and the formation of therapy-resistant metastases, most often in the lungs, brain, lymph nodes, and bones, making treatment extremely challenging than other BC subtypes (3, 6). Current treatment strategies for patients with TNBC include tumor resection, radiation, and chemotherapy.

Anthracyclines and taxanes are the most utilized cytotoxic drugs, as are platinum-containing drugs. Unfortunately, several of these treatments have substantial side effects, and because tumor cells are innately adaptable, chemoresistance has developed as a problem (7–10). As a result, new effective treatments against TNBC are necessary. While doxorubicin (DOX) is an effective treatment for a range of tumors, its cumulative, dose-related adverse effects restrict its clinical use (11). Myelosuppression, cachexia, cardiotoxicity, and skeletal muscle damage are only a few adverse effects (12–14). As a result, patients who might benefit from ongoing therapy have to switch to a less effective medication. Moreover, due to the intrinsic genetic instability of malignant cells, which may quickly develop resistance, it is often ineffective to use single-drug therapy to treat cancer, particularly aggressive forms such as TNBC (15). Thus, modulation of DOX therapy is urgently needed, given the lack of available therapeutic regimens for TNBC.

Consequently, a combination of drugs with distinct modes of action is more efficient and may be able to effectively treat the disease (15). In addition, combination treatment demonstrates more significant or at least comparable effectiveness with concentrations lower of every single agent and reduces the chance of drug resistance by simultaneously targeting several signal transduction pathways essential to carcinogenesis (16). Therefore, combination therapy is viewed as a viable strategy that may impact the future development of more successful therapeutic regimens for TNBC.

Adapalene (ADA), a 3^rd^ generation retinoid, is clinically used to treat acne vulgaris on a topical basis (17). In recent years, extensive research has analyzed the pharmacological properties of ADA and revealed its low toxicity and high stability in contrast to other retinoids (17). Studies report that ADA suppresses the growth of Hela, CC-531, and HepG2 cells and several malignancies both *in vitro* and *in vivo* (17–20). It has been reported that ADA treatment may increase ROS levels in cancer cells, which underlie the cancer cell killing activity of ADA (18). Based on the previous results, repurposing ADA for cancer treatment may be an effective therapeutic strategy.

In the present study, we investigated the anti-tumor potential of ADA in TNBC *in vitro* models and whether ADA can enhance the antitumor efficacy of doxorubicin in TNBC cells. The study’s rationale was that combining emerging treatment agents or repurposed pharmaceuticals with doxorubicin might increase susceptibility and the subsequent establishment of improved pharmacological combinations for treating TNBC (15, 21). Additionally, combined treatment will facilitate dosage reduction, reducing the toxicity associated with doxorubicin (11). We found that ADA reduced tumor cell growth and proliferation and significantly enhanced doxorubicin-induced growth inhibition of these cells and that ERK1/2 activity is involved in their synergistic effect. Our results indicate that treating TNBC with a combination of ADA and DOX may be more successful than DOX alone.

## Material and Methods

### Cell Culture and Reagents

Cayman Chemical (Ann Arbor, Michigan 48108 USA) supplied doxorubicin (DOX) (Cat. No. 1160) and Adapalene (ADA) (Cat. No. 13655). Cell culture media DMEM (Dulbecco’s Modified Eagle Medium), RPMI1640 (Roswell Park Memorial Institute Medium), & Fetal Bovine Serum (FBS) were procured from Gibco, Thermofisher Scientific USA. All the reagents used were of molecular grade or cell culture grade. TNBC cell lines (MDA-MB-231 and MDA-MB-468) and ER+ cell line MCF-7 were procured from the cell repository, National Centre for Cell Science (NCCS) Pune, India. Prof. Annapoorni Rangarajan (IISC, Bangalore, India) graciously provided the murine TNBC cell line 4T1. MDA-MB-231, MCF-7, and MDA-MB-468 cells were cultured in DMEM with 10% FBS and 1% penicillin-streptomycin. The murine TNBC cell line, 4T1, was cultured in RPMI-1640 media with FBS (10%) and penicillin-streptomycin (1%). At 37°C, the BC cell lines were cultured in a humidified CO2 incubator.

### Single Drug Cytotoxicity Assay

A cell viability assay was performed to determine the anti-tumor effect of ADA and DOX and generate a dose-effect curve required for the Chou-Talalay model for designing binary drug combinations (22, 23). In 96-well plates, BC cells (MDA-MB-468, 4T1, MCF-7, and MDA-MB-231) were cultured at 3 x 10^3^ cells/well. Seven distinct concentrations of DOX, ADA, or drug vehicle (DMSO), each with four replicates, were given the next day. After 72 hrs of incubation, the drug solutions were replaced, and new media with 5mg/ml MTT reagent was added using the MTT assay kit (24) (Cat No V-13154, Thermofisher Scientific). The growth inhibition was evaluated using the equation below (eq 1):


Eq. 1 $$\%\text{Inhibition}=\left[1-\left(\frac{\text{OD}\;\text{treated}\;\text{Cells}}{\text{OD}\;\text{vehicle}\;\text{control}\;\text{Cells}}\right)\right]\times 100$$

Where “OD treated cells” defines the mean absorbance of cells incubated with therapeutics, “OD vehicle control” implies the mean absorbance of cells treated with a complete cell culture medium containing 0.1 percent DMSO.

### Constant-Ratio Cytotoxicity Test for Binary Drug Combinations

The single-drug cytotoxicity assay of DOX and ADA in BC models laid the groundwork for the combination study. Six distinct equipotent DOX-ADA combinations were developed using the IC_50s_ of two drugs and evaluated in four repetitions in different cell lines. As proposed by Chou and Talalay, the DOX-ADA combinations were designed using the equipotent constant-ratio design (or diagonal technique), as shown in **Table 1** (22, 25). Following a 72-hr treatment period, the cytotoxic effects of drugs as individual agents or in combination were evaluated. As indicated before in Eq.1, each treatment’s percentage inhibition (effect) was calculated.

**Table 1:** Experimental Design and data summary of the dose-effect curve and Chou-Talalay parameters of doxorubicin and adapalene drug combinations against breast cancer cell lines after 72 hrs treatment period.

Cell Line	Doxorubicin(DOX)	Adapalene(ADA)	Fraction Affected(Fa)	Parameters			
				m	Dm	r	CI
MDA-MB-231	0.1 * IC50 0.25 * IC50 0.5 * IC50 0.75 * IC50 **IC50 (0.28 µM)** 1.25 * IC50	0.1 * IC50 0.25 * IC50 0.5 * IC50 0.75 * IC50 **IC50 (21.2µM)** 1.25 * IC50	0.16890 0.30804 0.56324 0.66716 0.77083 0.79985	1.1	0.30	0.98	0.74914 0.93978 0.73811 0.75161 0.63616 0.68357
MCF-7	0.1 * IC50 0.25 * IC50 0.5 * IC50 0.75 * IC50 **IC50 (0.14 µM)** 1.25 * IC50	0.1 * IC50 0.25 * IC50 0.5 * IC50 0.75 * IC50 **IC50 (25.4 µM)** 1.25 * IC50	0.27920 0.37246 0.54634 0.63769 0.68579 0.81200	0.85	0.15	0.98	0.48507 0.78334 0.76621 0.7869 0.84762 0.54222
MDA-MB-468	0.1 * IC50 0.25 * IC50 0.5 * IC50 0.75 * IC50 **IC50 (0.13 µM)** 1.25 * IC50	0.1 * IC50 0.25 * IC50 0.5 * IC50 0.75 * IC50 **IC50 (18.7 µM)** 1.25 * IC50	0.22899 0.27907 0.56781 0.68200 0.75925 0.84601	0.74	0.15	0.98	0.68975 1.27003 0.65398 0.58359 0.52201 0.37243
4T1	0.1 * IC50 0.25 * IC50 0.5 * IC50 0.75 * IC50 **IC50 (0.11 µM)** 1.25 * IC50	0.1 * IC50 0.25 * IC50 0.5 * IC50 0.75 * IC50 **IC50 (13.4µM)** 1.25 * IC50	0.25857 0.35729 0.58695 0.69310 0.70976 0.82156	0.84	0.11	0.96	0.5536 0.8538 0.65922 0.62439 0.76983 0.51982

m - Median; Dm - IC50; r- linear correlation coefficient CI - Combinational Index.

### Adoption of the Chou-Talalay Approach for Calculating the CI and DRI

The Combination Index (CI) value – a dimensionless variable used to identify and quantify the pharmacological interaction was computed by the CompuSyn software application, built on the combination Index Equation (Eq 2). When the CI value equals 1, an additive impact is obtained. Synergistic interaction is observed when the CI < 1 and antagonistic interaction when the CI > 1.


Eq. 2 $${\left(\text{CI}\right)}^{2}=\frac{{\left(\text{D}\right)}_{1}}{{\left(\text{Dy}\right)}_{1}}+\frac{{\left(\text{D}\right)}_{2}}{{\left(\text{Dy}\right)}_{2}}\;=\frac{{\left(\text{D}\right)}_{1}}{{\left(\text{Dm}\right)}_{1\;}[\text{fa }/{\left(1-\text{fa}\right]}^{1/\text{m}1}}\;+\;\frac{{\left(\text{D}\right)}_{2}}{\left(\text{Dm}{)}_{2}\right)\;[\text{fa }/{\left(1-\text{fa}\right]}^{1/\text{m}2}}$$

Where (Dy)1 is the concentration of drug 1 that alone reduces cell viability by y percent, (Dy)2 is the drug 2 concentration that alone reduces cell viability by y percent, and (D)1 and (D)2 are the concentrations of drug 1 (D1) and drug 2 (D2) taken together that reduce cell viability by y percent. The values of (Dy)1 and (Dy)2 may simply be obtained by rearranging the Median-Effect Eq (2), as shown in Eq. 3


Eq. 3 $$D=Dm\;{\left[\frac{fa}{1-fa}\right]}^{1/m}$$

The dimensionless function, dose reduction index, or DRI, evaluates and indicates the magnitude by which the concentration of the individual agent in a drug combination may be lowered compared to the doses of each drug alone at a given fractional inhibition. It was generated automatically by the CompuSyn program for experimental drug combinations based on the DRI Equations (22), as shown in Eq. 4.


Eq. 4 $${\left(\text{DRI}\right)}_{1}=\frac{{\left(\text{Dy}\right)}_{1}}{\text{D}1}\;,{\left(\text{DRI}\right)}_{2}=\;\frac{{\left(\text{Dy}\right)}_{2}}{\text{D}2},\;{\left(\text{DRI}\right)}_{3}=\;\frac{{\left(\text{Dy}\right)}_{3}}{\text{D}3}\ldots \;\ldots \;\text{.etc}.$$

DRI greater than 1 implies a desirable dosage decrease, DRI less than 1 suggests a detrimental dose reduction and DRI equal to 1 indicates zero dose reduction (22).

### Proliferation Assay

After assessing pharmacodynamic interactions, we examined the time-dependent effects of the synergistic drug combination DOX and ADA on cell proliferation. Cells were plated at 3 x 10^3^ cells/well in a 96-well plate and treated with ADA or DOX alone or combined at a concentration below the IC_50_. The proliferation of cells was determined after 24–72 hrs of incubation, using the Vybrant Proliferation Kit (Cat no. V-13154, Thermo Fisher Scientific USA).

### Colony Formation Assay

The effect of ADA, DOX, and their combined impact on the colony formation of cells was analyzed to assess the synergistic interactions further. Cells were plated at 1000–1500 cells per well in six-well plates (26, 27). After 48 hrs, fresh media was added and supplemented with therapeutics. The assay was performed for 14 to 18 days. The medium with therapeutics was replenished every three days, and colonies were observed in the wells using an inverted microscope. Once substantial colonies were formed, they were fixed with 3.7% paraformaldehyde (in PBS), and crystal violet (0.05%) was used for staining. Images of the plates were taken, and colonies were counted using the ImageJ application. The experiment was repeated three times for each cell type and treatment combination.

### Wound Healing Assay

Next, we investigated the individual and combined effect of DOX, and ADA, on the migration of the highly invasive TNBC cell lines MDA-MB-231 and 4T1 using the wound healing assay kit (Cat. no. CBA-120, Cell Biolabs, Inc., USA). The assay was performed in a 24-well plate, with cells seeded at 70% confluency and allowed to attach overnight with implanted scratch inserts. The scratch inserts were gently removed after 24 hrs, and the cells were washed with PBS. Fresh media with therapeutics was added, and cell migration was assessed after 48 hrs of treatment. Cells were fixed in 3.7% paraformaldehyde and stained with Giemsa stain. The cells were imaged, and the movement of cells into the wound site was examined and quantified using ImageJ software (28).

### Mammosphere Formation Assay

MDA-MB-231 cells as a single-cell suspension were seeded (1 x 10^4^ cells/well) onto ultralow attachment 6-well plates in 2ml DMEM/F12 (Gibco, 11320033) supplemented with 1 x B27 supplement (Invitrogen, 17504044) and SingleQuot™ (Lonza, CC-4136) (Gibco, 11320033) (Corning, 3471) (29). The next day, cells were treated with DOX, ADA alone, or in combination and cultured for five to ten days, with the medium being added every three days. The spheres were imaged using a phase-contrast inverted microscope (Nikon).

### Measurement of Reactive Oxygen Species

Next, we analyzed the accumulation of ROS upon treatment with therapeutics. MDA-MB-231 cells were seeded in 12-well plates and treated with ADA, DOX, or both for 24 hrs. Following staining with 10 µM DCFH-DA (Sigma) for 30 minutes in the dark, the cells were imaged using FLoid™ Cell Imaging Station (Thermo Fisher Scientific). Also, following staining, cells were collected and the fluorescence intensity was determined using an Agilent Fluorescence Spectrophotometer (30).

### Rhodamine-123 Staining Assay

Rhodamine 123 (Rh 123) staining was used to evaluate the mitochondrial membrane potential. As mitochondria transition from a polarized to a depolarized state during apoptosis, dye leakage occurs, leading to a decrease in the fluorescence intensity of Rh 123. MDA-MB-231 cells were seeded onto 24 well plates and treated with DOX, ADA, or combination of both for 24 hrs. Cells were stained for 15 minutes at 37 ℃ in the dark with 10 µM Rh 123 and washed thrice with 1x PBS and imaged using FLoid™ Cell Imaging Station. Also, following staining, cells were collected in PBS and fluorescence intensity was determined using Agilent Fluorescence Spectrophotometer (31). In some tests, cells were pretreated for 2 hrs with 5 mM N-acetyl cysteine (NAC) before exposure to the drugs.

### Western Blot Analysis

MDA-MB-231 cells were seeded in 6 cm dishes and treated with ADA, DOX, or both for 24 hrs. Following drug treatment, the cells were lysed with NP40 lysis buffer (Invitrogen, Thermo Fisher Scientific), supplemented with Halt™ Protease Inhibitor Cocktail using established protocols (32). Next, protein concentrations were measured using a BCA assay kit (PierceTM BCA Protein Assay Kit, Thermo Scientific Cat. No. 23227). Electrophoresis on SDS-polyacrylamide gels and electroblotting onto polyvinylidene difluoride membranes were used to separate the protein lysate. For 1.5 hrs at room temperature, 5% BSA was utilized for blocking. Specific primary antibodies against p-Erk1/2 (CST, Cat No. 4370, 1:2000), t-Erk1/2 (CST, Cat No. 4695 1:1000), PARP (CST, Cat No. 9542 1:1000), c-PARP (CST, Cat No. 5625, 1:1000), caspase-3 (CST, Cat No. 14220 1:1000), c-caspase-3 (CST, Cat No. 9664, 1:1000), caspase-9 (CST, Cat No. 9508, 1:1000), and c-caspase-9 (CST, Cat No. 52873, 1:1000) were used to probe protein bands. The binding of the primary antibody was detected using a secondary antibody coupled to horseradish peroxidase and visualized using an ECL kit (Bio-Rad, Hercules, CA). The immunoreactive protein bands were examined and normalized using GAPDH (CST, Cat No. 2118, 1:1000) as the loading control using ImageJ software.

### Annexin V Assay

Cells were grown in 12-well culture plates and treated with ADA, DOX, or both for 24 and 48 hrs. Next, floating and adherent cells were harvested and washed twice with ice-cold PBS. The washed cell samples were resuspended in 500 μl binding buffer containing 3 μl Annexin-V for 10 min and 2 μl 7-AAD for 15 min in the dark and, subsequently, evaluated for apoptosis (33, 34). Flow cytometry was performed at the Department of Biotechnology, National Institute of Technology, Rourkela Odisha, India, on a BD Accuri™ C6 Flow Cytometer. Apoptotic events were expressed as the percent of sub-G1 cells or the percent of apoptotic cells (combining early apoptotic Annexin V+/7-AAD − and late apoptotic Annexin V+/7-AAD+ cells).

### Cell Cycle Analysis

MDA-MB-231 cells were seeded at 50% confluency in 12-well plates and allowed to adhere overnight and serum-starved for cell cycle synchronization. Next, the cells were treated with DOX, ADA, or both for 24 and 48 hrs. Cells were trypsinized and fixed in 75% ethanol following treatment. After washing the cells, PI (0.5 mg/ml) and RNase A (10 mg/ml) was used to stain and assess the effect on the cell cycle. Prior to flow cytometry, cells were filtered using a 70 µm cell strainer. Flow cytometry was performed at the Department of Biotechnology, National Institute of Technology, Rourkela Odisha, India, on a BD Accuri ™ C6 Flow Cytometer (35).

### Statistics

IC_50_ values of compounds were calculated using non-linear regression analysis in GraphPad Prism. The statistical significance was analyzed using the one-way or two-way ANOVA in GraphPad Prism V 8.43, followed by Tukey multiple comparisons test. P < 0.05 was considered significant.

## Results

### Single Drug Cytotoxicity Assay

**Figure 1:**
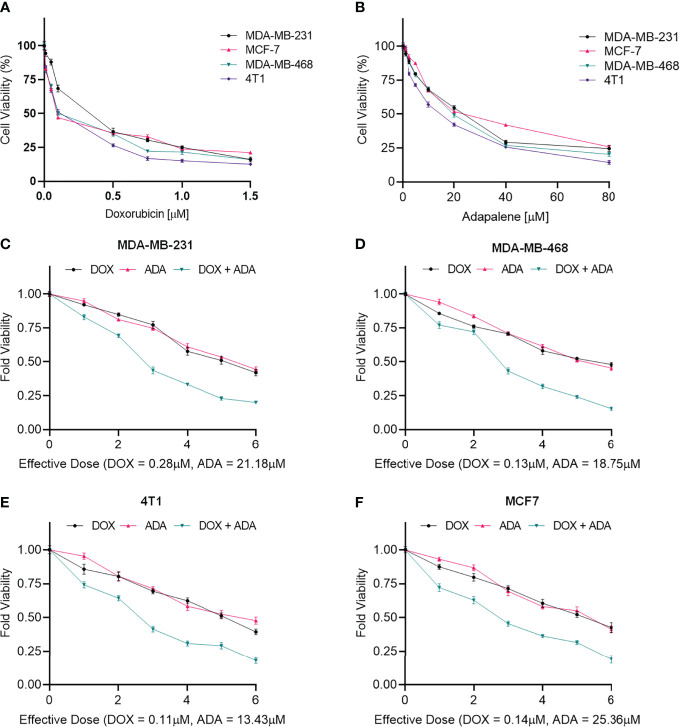
Doxorubicin and adapalene inhibited the growth of BC cells. Cell viability assay of BC cells treated with **(A)** Doxorubicin and **(B)** Adapalene. DOX and ADA both inhibited tumor cell growth in a dose-dependent manner. GraphPad prism was used for the calculations of the IC_50_ values. Treatment with combination of DOX and ADA showed an enhanced reduction in cell viability of **(C)** MDA-MB-231, **(D)** MDA-MB-468, **(E)** 4T1 and, **(F)** MCF-7 cells. When ADA and DOX were used together, the cell viability was significantly reduced, demonstrating that the drugs had advantageous pharmacodynamic interactions with one another.

MTT assay was carried out to evaluate the cytotoxicity of DOX and ADA alone against BC cell lines, and Graph-Pad prism v8 was used to produce dose-effect curves and obtain IC_50_ values for DOX and ADA (**Figures 1A, B**). DOX and ADA were both cytotoxic to all breast cell lines dose-dependently. The IC_50_ of DOX in MDA-MB-231, MCF-7, MDA-MB-468, and 4T1 was 0.28 µM, 0.14 µM, 0.13 µM and 0.11 µM respectively. DOX demonstrated high cytotoxicity in TNBC murine cell line 4T1. ADA showed an IC_50_ of 21.18 µM, 25.36 µM, 18.75 µM, and 13.43 µM in MDA-MB-231, MCF-7, MDA-MB-468, and 4T1, respectively. Based on the IC_50_ values, we designed an experimental setup for combination therapeutic evaluation.

### Cytotoxicity of Binary Drug Combination

**Figure 2:**
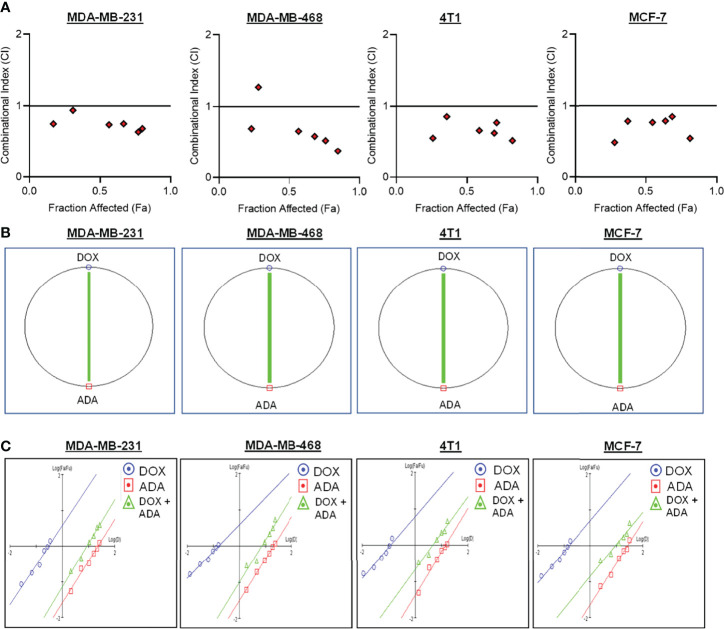
Doxorubicin and adapalene show synergistic pharmacodynamic interactions in BC models. **(A)** Combination Index (CI) plots of MDA-MB-231, MDA-MB-468, 4T1 and MCF-7 cells. The CI plots showed significant synergism between ADA and DOX in TNBC and ER+ MCF-7 cells. **(B)** Polygonograms of MDA-MB-231, MDA-MB-468, 4T1 and MCF-7 cells. **(C)** Median Plots of MDA-MB-231, MDA-MB-468, 4T1 and MCF-7 cells.

The single-drug cytotoxicity assay fulfilled the Chou-Talalay method’s criteria for commencing the *in vitro* pharmacodynamic drug interaction evaluation. We designed a constant-ratio combination approach or diagonal design. Cell viability was evaluated after 72 hrs of treatment (**Figures 1C–F**) The combination of DOX and ADA showed an enhanced reduction in cell viability of BC cells at very low doses, demonstrating positive drug-drug interactions of DOX and ADA. CompuSyn software was further utilized to calculate and quantify the CI, DRI values, and dose-inhibition curve parameters (**Table 1**). For MCF-7, MDA-MB-468, and 4T1, a flat sigmoidal (m < 1) curve was observed with an r-value (linear correlation coefficient) of approximately 0.97. MDA-MB-231 cells had a sigmoidal curve (m > 1) with approx. 0.99 for r. Also, the CompuSyn-calculated CI values for experimental points could achieve synergistic interactions as demonstrated with the CI less than 1 at precise combinations (**Table 1** and **Figure 2A**). The median-effect plots of drug combinations are shown in (**Figure 2C**).

### CompuSyn Software Simulation

A simulation algorithm was constructed using the median effect and combination index equations and the automation features of the CompuSyn software to simulate the estimated CI and DRI values at different fa levels. The simulated CI at different affected fraction levels was significantly synergistic, further validating *in vitro* results. The CompuSyn program also generated the Fa-Log CI plot, Fa-DRI plot, and isobolograms for each drug combination (**Supplementary Material**: CompuSyn reports). The simulated CI and DRI values at 50%, 75%, 90%, and 95% fraction affected are shown in (**Table 2**). Polygonograms at 50% fraction impacted levels were created to visually compare the kind and magnitude of drug interactions (**Figure 2B**). The solid line denotes synergistic interaction, the dashed line represents antagonistic interaction, and the thickness of the line indicates the degree of synergism or antagonism. Based on the simulated CI and DRI, it was further validated that all tested combinations exhibited synergistic interactions of varying magnitudes of inhibition, indicating that ADA acts in a synergetic manner with DOX.

**Table 2:** Summary of CompuSyn simulated CI and DRI values for Doxorubicin and Adapalene combination in breast cancer cell lines at 50%, 75%, 90%, and 95% growth inhibition.

Cell line	Drug CombinationDOX (D) + ADA (A)	CI Values at Inhibition of				DRI Values at Inhibition of			
		50%	75%	90%	95%	50%	75%	90%	95%
MDA-MB-231	D + A	0.75	0.70	0.65	0.62	D = 2.64 A = 2.66	D= 2.77 A= 2.80	D= 3.22 A= 2.89	D= 3.44 A= 3.03
MDA-MB-468	D + A	0.65	0.52	0.44	0.40	D= 3.26 A= 2.85	D= 5.67 A= 2.90	D= 9.87 A= 2.95	D= 14.38 A= 2.98
4T1	D + A	0.64	0.65	0.67	0.71	D=3.13 A=3.04	D= 3.86 A=2.54	D= 4.76 A= 2.13	D= 5.49 A= 1.88
MCF-7	D + A	0.66	0.75	0.8	1.0	D= 3.13 A= 2.90	D= 3.31 A= 2.19	D= 3.5 A= 1.65	D= 3.63 A= 1.37

### ADA Inhibits Proliferation and Enhances Sensitivity to DOX in TNBC Cells

**Figure 3:**
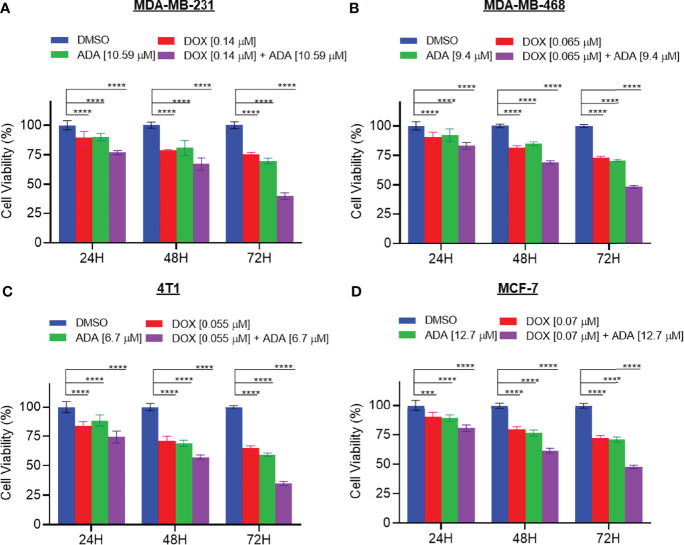
The combination of doxorubicin and adapalene synergistically reduces tumor cell proliferation. The combination of DOX and ADA inhibited proliferation of **(A)** MDA-MB-231, **(B)** MDA-MB-468, **(C)** 4T1 and, **(D)** MCF-7 in a synergistic manner. Data are mean ± SD. p-values were determined by two-way ANOVA followed by Tukey’s multiple comparisons test (***p < 0.001; ****p < 0.0001). Significant reduction in cell viability was observed when treated in a time-dependent manner with combined treatment of DOX and ADA showing maximal effect. Data are representative of at least three independent experiments.

We further evaluated the synergetic drug combination of DOX and ADA in a time-dependent manner. We proceeded with a single synergistic drug combination below individual IC_50_ among several drug combinations designed earlier. The cell viability was analyzed at 24, 48, and 72 hrs using the Vybrant cell proliferation kit (Invitrogen, Thermofisher, USA) following the manufacturer’s protocol. Combinatorial treatment significantly reduced cell proliferation compared to single-agent treatment (**Figures 3A–D**). The results demonstrate that DOX and ADA in combination enhance the anti-proliferative effect of each other synergistically. Moreover, the sensitivity of TNBC cells towards DOX significantly increased upon co-treatment with ADA. The trend was seen in all three time periods and all the four cell lines of BC.

### DOX and ADA Combination Disrupts Colony Formation and Migration of TNBC Cells

**Figure 4:**
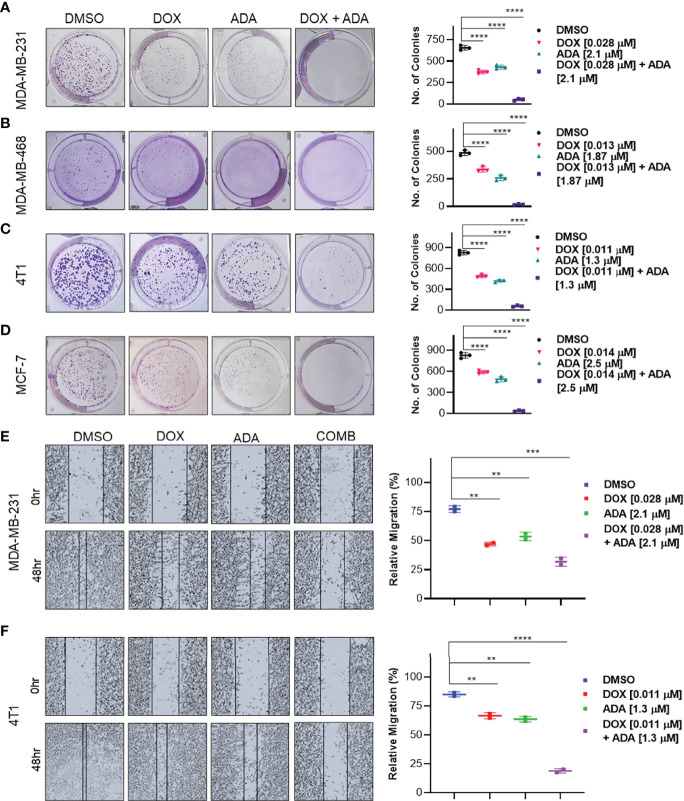
The combination of doxorubicin and adapalene inhibits colony formation and migration potential of TNBC cells. Representative images and quantification of colony formation assay data of **(A)** MDA-MB-231, **(B)** MDA-MB-468, **(C)** 4T1 and, **(D)** MCF-7 cells are treated with drug vehicle (DMSO), DOX, ADA, or a combination of DOX & ADA. The right panels show the quantification of colonies formed under each treatment condition described in the left panels. Data are mean ± SD. P values were determined by one-way ANOVA followed by Tukey’s multiple comparisons test. Representative images and quantification of migration assay data of **(E)** MDA-MB-231, **(F)** 4T1. The right panels show the relative migration under control, single treatment and combination of DOX and ADA described in the left panels. Data are mean ± SD. P values were determined by one-way ANOVA followed by Tukey’s multiple comparisons test. Data are representative of at least three independent experiments. (**p < 0.001; ***p < 0.005, ****p < 0.0001).

Experiments with colony formation in BC cell lines were utilized to validate further the anti-tumor activity and synergistic interactions of ADA with DOX. While treatment with DOX and ADA alone resulted in a decrease in colony formation, combined treatment with DOX and ADA resulted in a considerable reduction in colony formation compared to individual drug treatments. Further study found that the number of colonies in each treated cell line was equivalent when treated alone; however, the number of colonies was significantly reduced when treated in combination. The study also demonstrated that ADA as a single agent reduces tumor cell growth, inhibiting the colony formation of breast tumor cells (**Figures 4A–D**).

Cancer cells must infiltrate the ECM and undergo the multistep phenomenon of metastasis to colonize distant organs. As a result, blocking cell migration is a promising approach to prevent metastasis. This study aimed to determine the effect of the combination of DOX and ADA on tumor cell motility. CytoSelect™ 24-Well Wound Healing Experiment Kit was used to perform the assay in 24 well plates. MDA-MB-231 and 4T1 cells were treated for 48 hrs with DOX or ADA alone or in combination, and migration of cells was assessed using ImageJ software. The combination of DOX and ADA significantly reduced migration compared to control cells or cells treated with DOX or ADA alone (**Figures 4E, F**).

**Figure 5:**
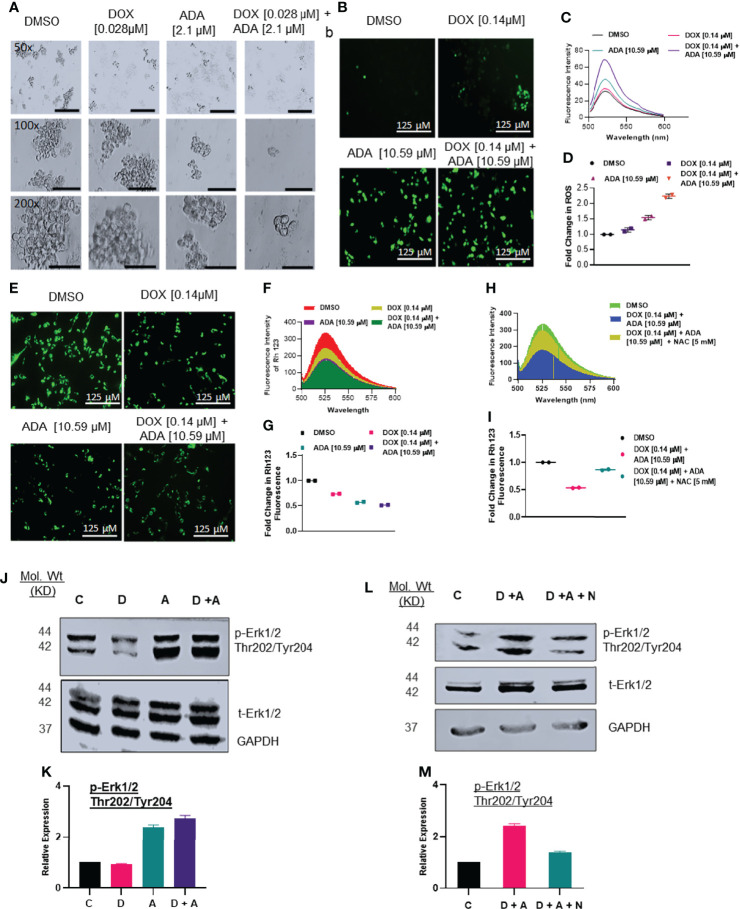
Doxorubicin and adapalene affect the anchorage-independent growth of MDA-MB-231 cells and enhance apoptosis. **(A)** Representative images of the spheroid assay. Treatment with the combination of DOX and ADA significantly reduced the growth of TNBC cells in ultra-low attachment plates and reduced mammosphere size (Bar: 500 μm (50X), 200 μm (100X and 200X). **(B)** DCF-DA staining, **(C)** fluorescence intensity & **(D)** fold change in ROS levels in MDA-MB-231 cells treated with ADA or DOX alone or in combination. **(E)** Rhodamine 123 staining, **(F)** fluorescence intensity & **(G)** fold change in Rh123 staining levels in MDA-MB-231 cells treated with ADA or DOX alone or in combination both showing decrease in mitochondrial membrane potential upon treatment. **(H)** Fluorescence intensity & **(I)** fold change in Rh123 upon treatment with DOX-ADA and DOX-ADA + NAC [5µM]. **(J, K)** Western blots of p-Erk1/2 (Thr202/Tyr204) on MDA-MB-231 with DOX/ADA or the combination of both. **(L, M)** Western blots of p-Erk1/2 (Thr202/Tyr204) on MDA-MB-231 with DOX-ADA or DOX-ADA + NAC [5µM]. Data are representative of at least two independent experiments. *C, control; D, doxorubicin; A, adapalene; A + D, adapalene + doxorubicin; A + D + N, adapalene + doxorubicin + NAC*.

Mammosphere assays are widely used *in vitro* to identify prospective cancer-initiating stem cells that can propagate clonally to form spheres in free-floating conditions (36). We evaluated the effect of DOX and ADA on spheroid formation. The combination of DOX and ADA significantly repressed the anchorage-independent growth of MDA-MB-231 cells and suppressed mammosphere formation, as shown in (**Figure 5A**). These results further support that combined treatment with DOX and ADA has significant tumor-reducing activity in TNBC.

### Combined Treatment With Adapalene and Doxorubicin Enhanced ROS Production and Impaired Mitochondrial Function

Next, we sought to elucidate the mechanisms driving the synergistic action of ADA and DOX in BC cells. Previously, it was suggested that most anticancer drugs act by modulating oxidative stress and regulating apoptosis (37). We determined the intracellular ROS levels using DCF-DA following treatment with DOX, ADA, or both. The results indicated that ADA elevated ROS levels in MDA-MB-231 cells, further intensified when DOX was added (**Figures 5B–D**). Additionally, we observed that DOX has a minimal influence at the concentration used in our study on ROS levels compared with ADA but dramatically augments ROS levels when combined with ADA.

ROS production is associated with disrupting the mitochondrial membrane potential (MMP), a critical step in initiating apoptosis, which can be detected using the Rh 123 staining. Compared to untreated controls, the cells treated with ADA, DOX, or both exhibited low fluorescence intensities for Rh 123. The MMP was lowest for the cells treated with the combination of DOX and ADA (**Figures 5E–G**). Additionally, membrane disruption was rescued by pre-treatment with NAC (5mM) for 2 hrs prior to DOX and ADA co-treatment (**Figures 5H, I**). These findings imply that oxidative damage, which disrupts the mitochondrial membrane potential, may contribute significantly to the increased lethality observed in the combination of ADA and DOX treatment of MDA-MB-231 cells.

### Hyperactivation of Erk1/2 Upon Treatment With DOX and ADA Triggers Intrinsic Apoptosis

Intracellular ROS production or oxidative stress generated by various anticancer therapies is well known to play a vital role in induced apoptosis *via* signalling cascade regulation (38). We sought to investigate the effect of co-treatment of DOX and ADA on MAPK signalling. Combined treatment with ADA and DOX promoted hyperphosphorylation of Erk1/2 (**Figure 5J–K**). Notably, we showed that NAC (free radical scavenger) reduced ROS-driven phosphorylation of Erk1/2, supporting the involvement of stress induced by ADA in driving Erk1/2 phosphorylation (**Figure 5L–M**). These findings imply that ROS production is critical for the anti-tumor activity of ADA and its synergistic action with DOX.

**Figure 6:**
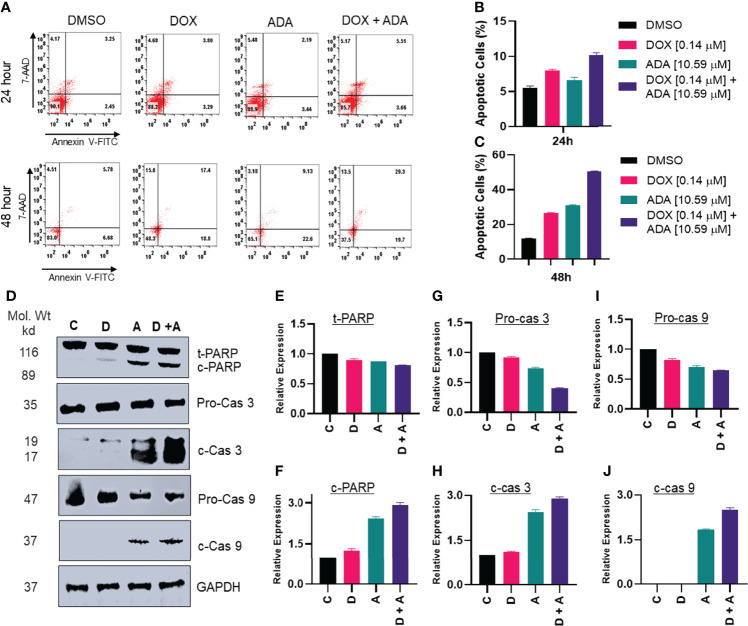
Adapalene and doxorubicin, in a synergistic manner, promote apoptosis. **(A–C)** Annexin V & &-AAD staining showed high apoptotic cells in plates treated with the combination of DOX or ADA or both after 24h or 48hr periods. **(D)** Western blots of expression levels of PARP, c-PARP, pro-caspase 3, c-caspase 3, pro-caspase 9 and c-caspase 9 on MDA-MB-231 with DOX/ADA or both for 24 hrs. Co-treatment showed enhanced PARP cleavage and activation of caspase 3 and caspase 9. C*, control (Treated with drug vehicle); D, doxorubicin; A, adapalene; A + D, adapalene + doxorubicin* **(E–J)** Relative expression plots of western blots.

Previously, it was shown that ERK1/2 phosphorylation is required for oxidative stress-induced apoptosis (38). Additionally, once apoptosis drivers are active due to mitochondrial membrane potential depletion, ERK1/2 may initiate caspase-mediated apoptosis. We sought to determine the expression of caspase 3, cleaved caspase 3, caspase 9, cleaved caspase 9, PARP, and cleaved PARP following treatment with DOX, ADA, or both. Combined treatment with ADA and DOX resulted in enhanced cleaved caspase 9, cleaved PARP, and cleaved caspase 3 (**Figures 6D–J**).

Additionally, we used Annexin-V and 7-AAD staining to determine the apoptosis-inducing capacity of ADA, DOX, or their combination. The flow cytometry study demonstrated that ADA promotes apoptosis in tumor cells and increases apoptosis upon combination therapy (**Figures 6A–C**). DOX also induced apoptosis, although the degree of apoptosis was much more significant in combination therapy than in single-agent treatment. Also, the necrotic cell population was high in cells treated with DOX for 48 hrs, while combination therapy reduced the necrotic cell population and enhanced apoptotic cells.

### Treatment With DOX and ADA Inhibits Cell Cycle Progression

**Figure 7:**
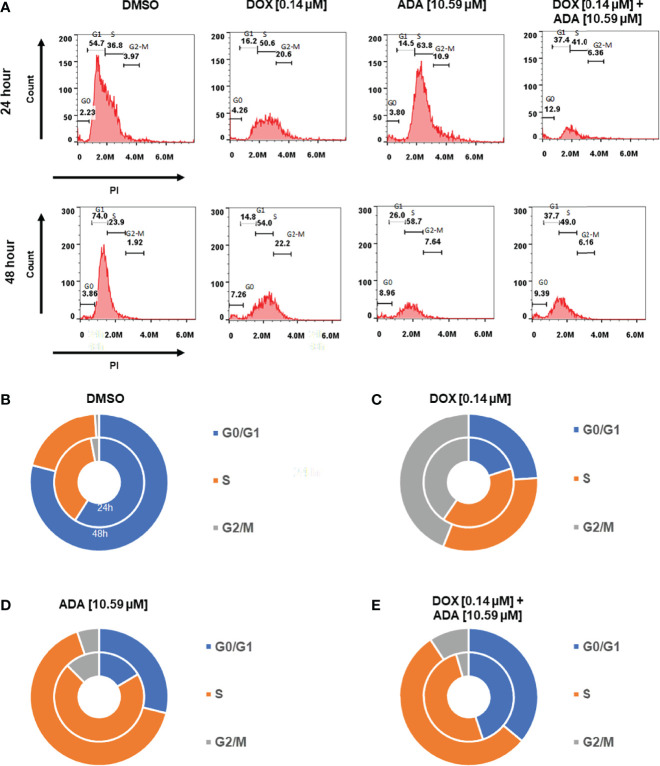
Doxorubicin and adapalene induce cell cycle arrest of MDA-MB-231 cells. **(A–E)** ADA upon treatment showed S-phase arrest of MDA-MB-231 cells, while DOX showed the arrest of MDA-MB-231 cells in the G2/M phase of the cell cycle. Upon Combination treatment with DOX and ADA, the arrest of cells enhanced in the s-phase.

**Figure 8:**
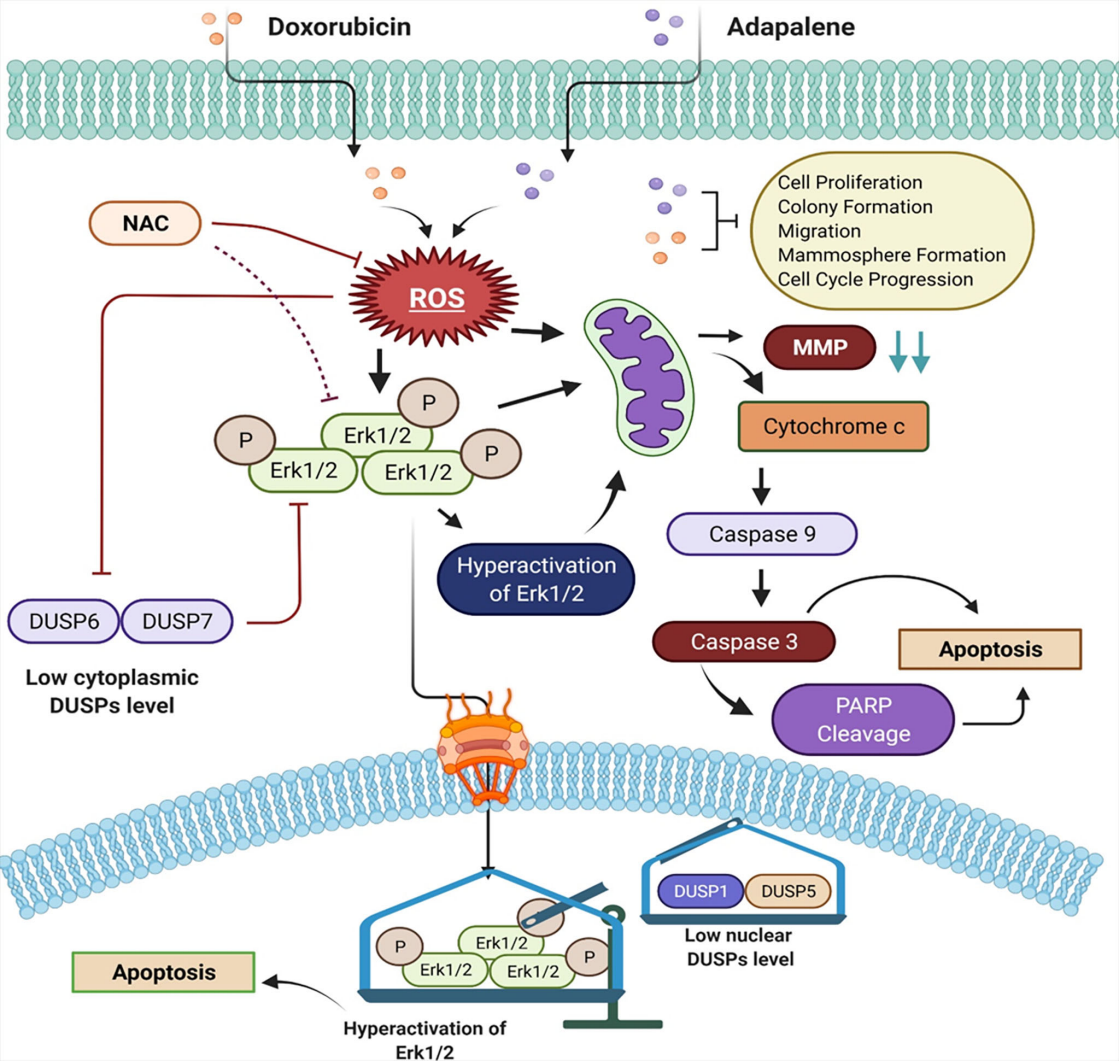
Schematic of the main findings of the study and possible mechanism of action of doxorubicin and adapalene in TNBC cells. The co-treatment with DOX and ADA resulted in enhanced ROS generation, leading to mitochondrial membrane potential disruption. Disruption of MMP triggers intrinsic apoptosis associated with PARP cleavage. Also enhanced ROS generation, triggers hyperactivation of Erk1/2 signalling, which further promotes apoptosis *via* mitochondrial death pathway.

Cells self-replicate *via* a process called the cell cycle. Since cell cycle arrest inhibits cancer cell proliferation, it may represent a critical method for cancer treatment. To evaluate whether cell cycle arrest contributes to the synergistic effects observed following combination treatment, the cell cycle profile of MDA-MB-231 cells was examined after 24 and 48 hrs of treatment with DOX, ADA alone, or in combination. Flow cytometry results demonstrated that ADA induced S-phase cell cycle arrest in MDA-MB-231 while DOX promoted the arrest of MDA-MB-231 cells in the G2/M phase **Figure 7**. In combination, DOX and ADA enhanced the arrest of cells in the S-phase of the cell cycle **Figure 8**.

## Discussion

Combining targeted and conventional therapies are becoming a therapeutic standard for various haematological and solid cancers (15, 39). Due to toxicities and drug resistance, standard chemotherapeutic agents, such as doxorubicin, are restricted (40). Additionally, resistance to doxorubicin is prominent in breast cancer and is typically associated with a multidrug resistance phenotype (41, 42). As a result, it is crucial to design combination treatment regimens comprising doxorubicin and chemosensitizer agents that improve rather than diminish its anticancer effectiveness while reducing its side effects. Recently, attention has been drawn to the combination treatment with conventional chemotherapeutic agents (15, 43). In this study, we demonstrated that the combined effect of ADA and DOX substantially increased apoptosis in TNBC cells. Increased intracellular ROS generation, Erk1/2 activation, and apoptosis were the primary mediators of this synergistic effect. Additionally, we observed a synergistic inhibitory effect of ADA and DOX on colony formation, cell migration, mammosphere formation, and cell proliferation.

Cancer cells produce and retain a larger proportion of reactive oxygen species (ROS) than normal cells. Increased reactive oxygen species (ROS) render tumor cells more sensitive to ROS-generating substances (37, 44). Studies have indicated that increasing ROS production in cancer cells inhibits tumor development and induces apoptosis. As a result, facilitating ROS is a promising therapeutic approach for cancer (21, 45, 46). Several chemotherapeutic agents have produced anticancer effects by activating the intrinsic apoptotic signalling. Mitochondria are both producers and targets of reactive oxygen species (ROS) (47). Excessive ROS generation may result in the loss of mitochondrial membrane potential (MMP), allowing apoptotic effectors to escape (48). The mitochondria-mediated apoptosis pathway is dependent on cytochrome c release into the cytosol. It is required to form the apoptosome and activation of caspase 9, which leads to caspase 3 and caspase 7 activations. Caspase 3 and 7, the executors of the caspase family, break PARP, a characteristic of apoptosis. (49, 50). Our study demonstrates that ADA, either alone or combined with DOX, increases ROS generation, inducing MMP disruption. Pre-treatment with NAC reduced MMP disruption upon co-treatment of DOX and ADA. Additionally, we found that the inhibition of MMP by ADA and DOX activates the mitochondrial intrinsic apoptotic pathway *via* caspase 9 and caspase 3 activation and PARP cleavage, as shown in (**Figure 8**) diagrammatic summary. As a result, we infer that combination therapy enhances ROS generation, mitochondrial malfunction, and eventually caspase-dependent death in TNBC cells.

The serine/threonine-protein kinase, ERK, is a member of the mitogen-activated protein kinases (MAPKs) family. Protein kinase mutations and dysregulation are involved in the pathogenesis of human illness and serve as a platform for developing therapeutic agonists and antagonists. Depending on the type of cell and stimulus, activation of ERK has been demonstrated to trigger apoptosis. Numerous anticancer drugs have been shown to activate ERK in various cancer cell types. Increased ROS accumulation linked with oxidative stress stimulates the Ras/Raf/ERK signalling pathway. The Ras/Raf/ERK pathway is activated in conjunction with the intrinsic apoptotic pathway, defined by the release of cytochrome c from the mitochondria and activation of the initiator caspase 9 (51, 52).

Herein, we found that ADA activates Erk1/2 in TNBC cells *via* increased ROS production, and this activation was rescued by pre-treatment with NAC. Additionally, we observed that combining ADA with DOX resulted in increased levels of (activated) p-ERK, unravelling the reason behind the synergistic effects of the DOX and ADA combination. Previously, it has been reported that drugs inducing ROS-mediated ERK activation sensitize anticancer therapies (53). For example, curcumin enhances the anticancer activity of cisplatin in bladder cancer cell lines *via* activating ERK1/2 through ROS-mediated signalling (54). Co-treatment with curcumin and cisplatin triggered activation of p53, apoptosis, and downregulation of survival proteins, which were reduced by NAC (a ROS scavenger) and U0126 (a MEK inhibitor). Also, curcumin and cisplatin caused apoptosis in bladder cancer cells *via* ROS-mediated activation of ERK1/2 (55).

Consequently, the growing concept that ERK1/2 may trigger cell death and the strategy/compound for enhancing pro-apoptotic ERK activity may provide a new therapeutic window for malignancies with oncogenic ERK signalling pathway activations. Chronic ERK activation, for instance, increases cell death in several cancer cell lines (55, 56). Additionally, altering ERK activity in a particular subcellular compartment may promote tumor cell death. (55). Notably, ACA-28 preferentially kills cancer cells with high ERK activity, while ACAGT-007 has shown better potency and selectivity against high-ERK melanoma cells *in vitro*. For ERK-induced apoptosis as an anti-cancer therapy, it is necessary to overcome the issue of selectively inducing apoptosis in cancer cells with abnormal ERK activity while preserving the survival of normal cells. In addition, the development of therapeutic molecules or the repurposing of existing drugs with the ability to enhance ROS-dependent ERK activation is a viable therapeutic approach for TNBC (55).

Understanding the molecular mechanisms behind the anti-tumor activity of ADA and its pharmacodynamic interactions with DOX may help design ADA-DOX combination therapy. As previously documented, ADA triggers several pro-apoptotic responses that result in apoptosis in various tumor types (17). ADA promotes apoptosis in colorectal cancer cells *via* activating the caspase-3 and Bax/Bcl-2 pathways. Recent studies showed that ADA-mediated tumor growth inhibition occurs due to DNA damage. Melanoma cells treated with ADA produced increased levels of DNA damage marker, ϒ-H2AX (57). Apart from its increased ability to inhibit proliferation and promote apoptosis, it may have several physiological advantages over standard retinoic acid derivatives. ADA exhibits more anti-inflammatory effects *in vitro* and *in vivo* than other retinoids due to its suppression of lipoxygenase pathways (58). ADA is five times more stable to light than natural retinoids due to its chemical makeup.

Additionally, ADA has a safer profile than other retinoids with oral 5 g/kg LD_50_ in rats and mice, significantly higher than 9-cis-retinoic acid + (58, 59). Also, high dosages of ADA administered orally have no adverse effects on the neurologic, hematologic, cardiovascular, or respiratory systems (17). The previous and present study findings demonstrate that ADA is a potent anti-tumor agent.

To summarise, we report that ADA is a potent anticancer agent and improved the anticancer activity of DOX *via* ROS-mediated hyperactivation of the Erk1/2 signalling pathway. These findings shed light on the molecular pathways through which ADA and DOX interact and imply that such combination therapy may become a more successful treatment for TNBC.

The validation of our results is restricted to the experimental methodology described, and unquestionably, additional screening studies are required to determine the optimal combination regimens. For example, the successive addition of drug combinations could drastically reverse the net effects. In addition, we suggest conducting additional *in vitro* pharmacodynamic interaction analyses based on a non-constant experimental design so that more potent combinations with a more favourable DRI can be achieved. In addition, further research might be done to determine the impact of the observed synergistic combinations on *in vivo* BC models and other cancer hallmarks. Our results indicate that ADA and DOX may have therapeutic potential for TNBC. The results of this study should be validated in relevant preclinical models of TNBC to determine their clinical importance.

## Data Availability Statement

The original contributions presented in the study are included in the article/**Supplementary Material**. Further inquiries can be directed to the corresponding author.

## Author Contributions

MM designed and supervised the study. UM performed the experiment, collected, analyzed the data, and wrote the manuscript. MM, NW, IM, MH, and MA performed the analysis and critically revised the manuscript. All authors read and approved the manuscript.

## Funding

This work was funded by the JK Science Technology & Innovation council DST, Govt. of JK, JK India with grant No. JKST&IC/SRE/885-87 to MA.

## Conflict of Interest

The authors declare that the research was conducted in the absence of any commercial or financial relationships that could be construed as a potential conflict of interest.

## Publisher’s Note

All claims expressed in this article are solely those of the authors and do not necessarily represent those of their affiliated organizations, or those of the publisher, the editors and the reviewers. Any product that may be evaluated in this article, or claim that may be made by its manufacturer, is not guaranteed or endorsed by the publisher.
