# Supplementary material for: Adapalene and Doxorubicin Synergistically Promote Apoptosis of TNBC Cells by Hyperactivation of the ERK1/2 Pathway Through ROS Induction
Source: Front Oncol. 2022 Jul 6;12:938052. doi: 10.3389/fonc.2022.938052 (PMC9298514; doi:10.3389/fonc.2022.938052)
Supplement: Supplementary file 1 [file DataSheet_1.pdf]

Adapalene synergistically with doxorubicin promotes apoptosis of TNBC Cells by hyperactivation of the ERK1/2 pathway through ROS induction

Umar Mehraj[1], Irfan Ahmad Mir[2], Mahboob ul Hussain[2], Mustafa Alkhanani[3], Nissar Ahmad Wani[4], Manzoor Ahmad Mir[1*]

[1] Department of Bioresources, School of Biological Sciences, University of Kashmir, Srinagar, 190006, J&K India.
[2] Department of Biotechnology, School of Biological Sciences, University of Kashmir, Srinagar, 190006, J&K India.
[3] Department of Pharmacy Practice, College of Pharmacy AlMaarefa University Riyadh-13713, KSA
[4] Department of Biotechnology, School of Life Sciences, Central University of Kashmir, Ganderbal, 191201, J&K India.

***Corresponding author:** drmanzoor@kashmiruniversity.ac.in, (M.A.M.)

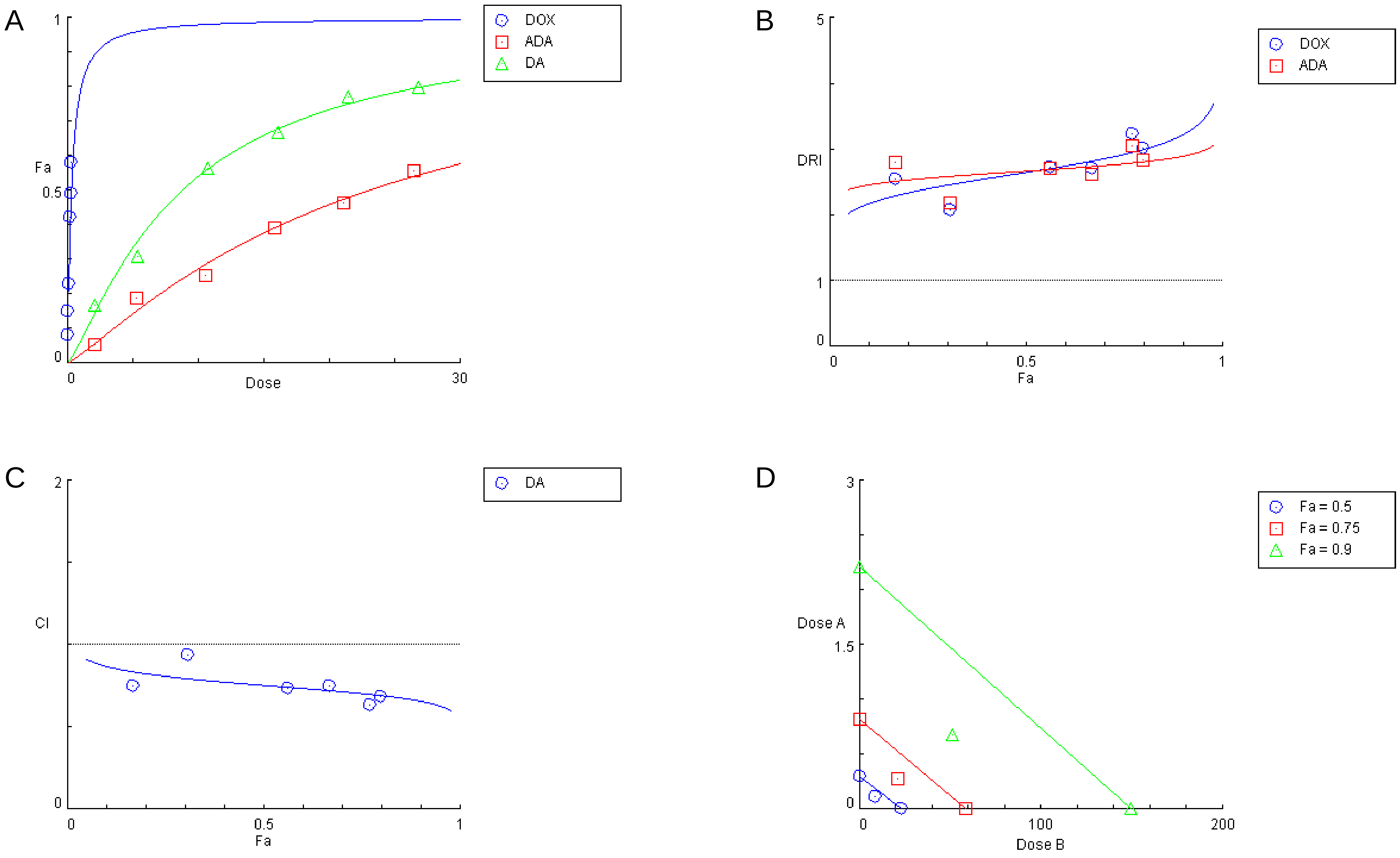

Figure S1: CompuSyn calculated parameters of binary drug combination at actual experiment points a. Dose-Effect Curve b. DRI plot c. Fa-CI plot d. Isobologram

Cell Line-MDA-MB-231

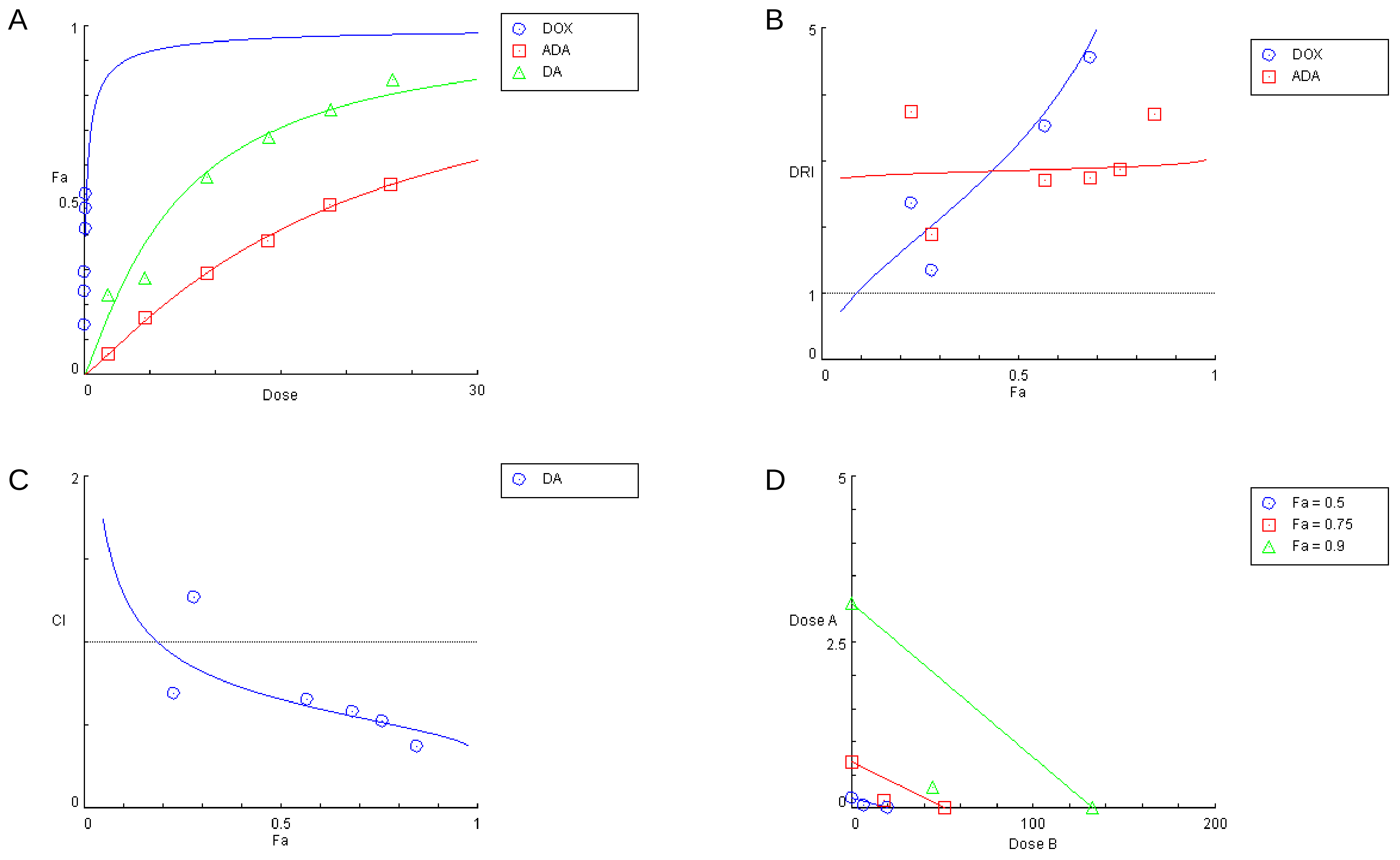

Figure S2: CompuSyn calculated parameters of binary drug combination at actual experiment points a. Dose-Effect Curve b. DRI plot c. Fa-CI plot d. Isobologram

Cell Line: MDA-MB-468

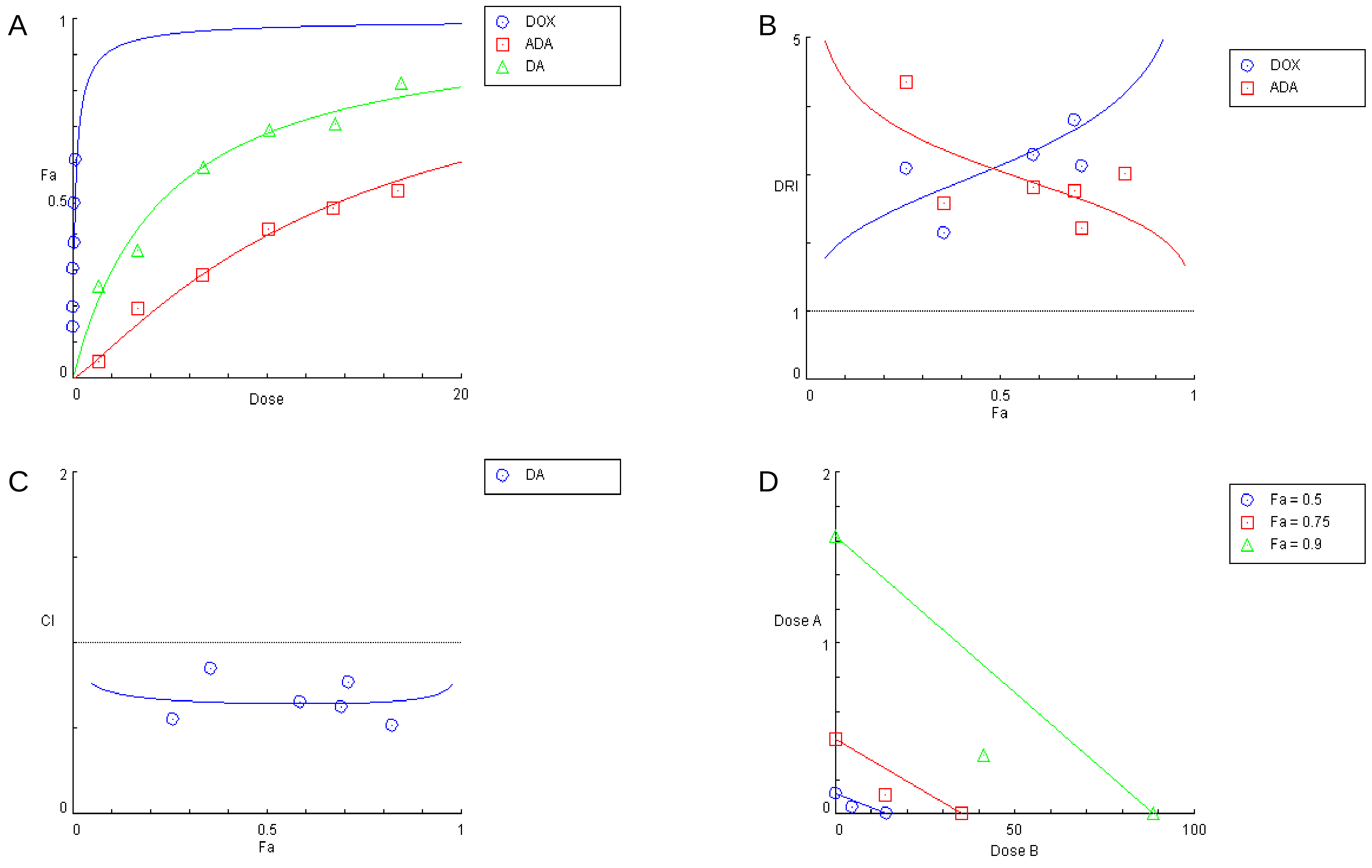

Figure S3: CompuSyn calculated parameters of binary drug combination at actual experiment points a. Dose-Effect Curve b. DRI plot c. Fa-CI plot d. Isobologram

Cell Line:4T1

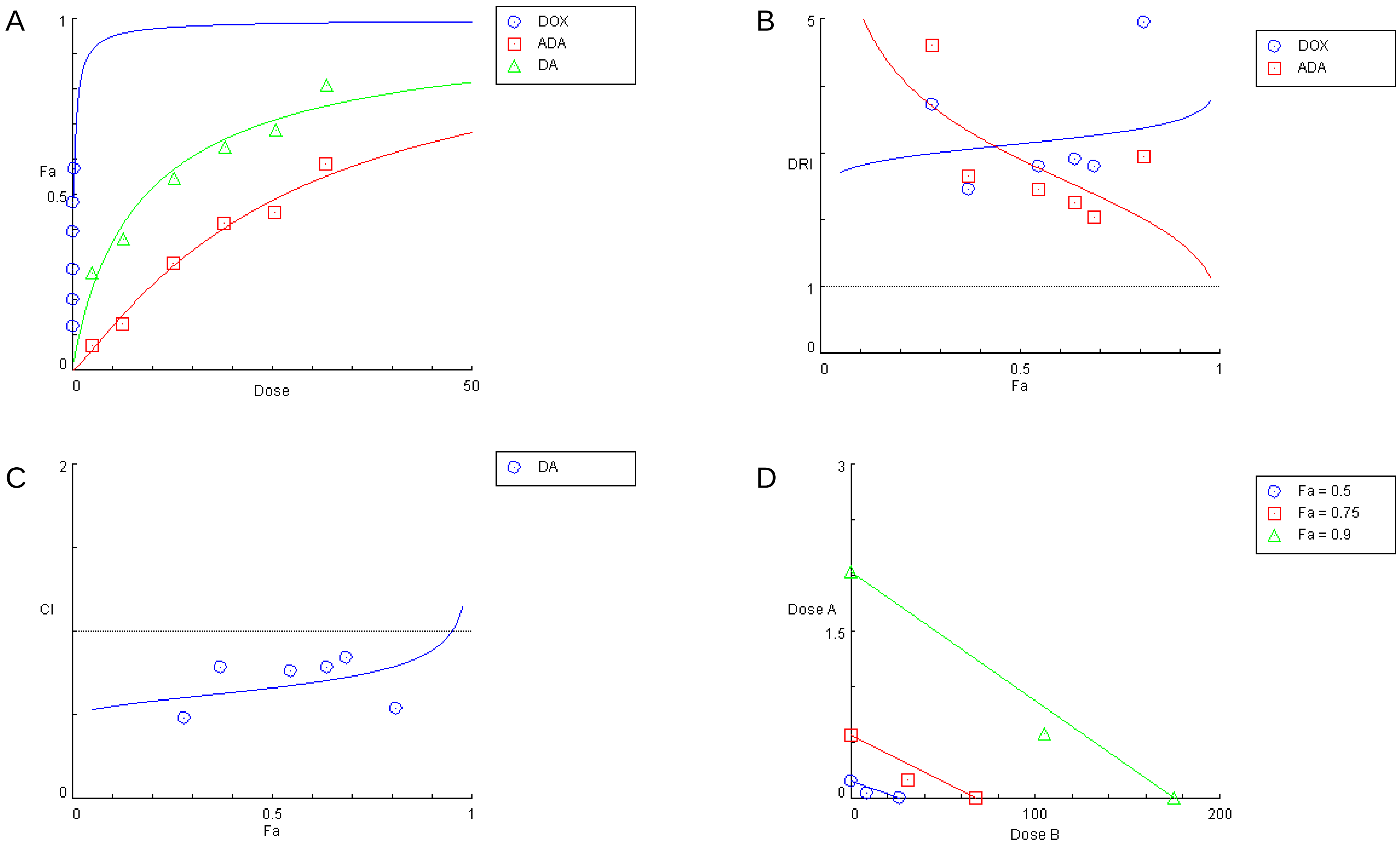

Figure S4: CompuSyn calculated parameters of binary drug combination at actual experiment points a. Dose-Effect Curve b. DRI plot c. Fa-CI plot d. Isobologram

Cell Line:MCF-7

Table S1: List of antibodies used in the Study

Protein	Company	Catalogue number	Dilution
**GAPDH**	CST	2118	1:1000
**p-Ek1/2**	CST	4370	1:2000
**t-Erk1/2**	CST	4695	1:1000
**PARP**	CST	9542	1:1000
**Cleaved PARP**	CST	5625	1:1000
**Caspase-9**	CST	9508	1:1000
**Cleaved Caspase-9**	CST	52873	1:1000
**Caspase-3**	CST	14220	1:1000
**Cleaved Caspase-3**	CST	9664	1:1000

Table S2: Percentage of populations, events, %total, %parents of MDA-MB-231 cells treated with DMSO, DOX, ADA, and the combination of DOX and ADA for 24 hrs.

**DMSO 24h**					
POPULATION	Events	% total	% Parent	Mean FL4-A	Mean FL3-A
ALL EVENTS	10,000	100%	100%	-	-
P1	10,000	100%	100%	-	-
Q-UL	417	4.17%	4.17%	1,536.78	5,902.68
Q-UR	325	3.25%	3.25%	6,657.14	20,138.68
Q-LL	9,013	90.1	90.1	1,336.19	855.64
Q-LR	245	2.45%	2.45%	3,804.00	1,098.58

**DOX 24h**					
POPULATION	Events	% total	% Parent	Mean FL4-A	Mean FL3-A
ALL EVENTS	10,000	100%	100%	-	-
P1	10,000	100%	100%	-	-
Q-UL	468	4.68%	4.68%	1,369.18	4,448.45
Q-UR	380	3.80%	3.80%	7,078.91	18,863.79
Q-LL	8823	88.23%	88.23%	1,259.29	964.54
Q-LR	329	3.29%	3.29%	3,930.38	1,027.59

**ADA 24h**					
POPULATION	Count	% total	% Parent	Mean FL4-A	Mean FL3-A
ALL EVENTS	10,000	100%	100%	-	-
P1	10,000	100%	100%	-	-
Q-UL	548	5.48%	5.48%	1,419.37	5,864.21
Q-UR	219	2.19%	2.19%	10,235.00	36,375.52
Q-LL	8889	88.89%	88.89%	1,516.58	815.39
Q-LR	344	3.44%	3.44%	3,936.42	852.14

DOX+ ADA 24h					
POPULATION	Count	% total	% Parent	Mean FL4-A	Mean FL3-A
ALL EVENTS	10,000	100%	100%	-	-
P1	10,000	100%	100%	-	-
Q-UL	517	5.17%	5.17%	1,437.60	4,636.88
Q-UR	551	5.51%	5.51%	6,556.53	20,012.73
Q-LL	8566	85.66%	85.66%	1,251.81	1,035.30
Q-LR	366	3.66%	3.66%	3,684.04	1,149.91

Q-UL	Quadrant-Upper Left
Q-UR	Quadrant-Upper Right
Q-LL	Quadrant-Lower LEFT
Q-LR	Quadrant-Lower Right

Table S3: Percentage of populations, events, %total, %parents of MDA-MB-231 cells treated with DMSO, DOX, ADA, and the combination of DOX and ADA for 48 hrs.

DMSO 48h					
POPULATION	Events	% total	% Parent	Mean FL4-A	Mean FL3-A
ALL EVENTS	10,000	100%	100%	-	-
P1	4,722	100%	47.22%	-	-
Q-UL	213	4.50%	2.13%	1,488.96	5,519.40
Q-UR	273	5.80%	2.73%	12,344.62	29,938.63
Q-LL	3,919	6.70%	39.19%	1,203.77	583.32
Q-LR	316	83.00%	3.16%	4,090.66	606.71

DOX 48h					
POPULATION	Events	% total	% Parent	Mean FL4-A	Mean FL3-A
ALL EVENTS	10,000	100%	100%	-	-
P1	4,358	100%	43.58%	-	-
Q-UL	681	15.60%	6.81%	1,582.46	4,774.66
Q-UR	758	17.40%	7.58%	6,480.14	17,339.68
Q-LL	2100	48.20%	21.00%	1,476.33	1,054.91
Q-LR	819	18.80%	8.19%	4,041.42	1,158.96

ADA 48h					
POPULATION	Count	% total	% Parent	Mean FL4-A	Mean FL3-A
ALL EVENTS	10,000	100%	100%	-	-
P1	2,801	100%	28.01%	-	-
Q-UL	89	3.18%	0.89%	1,807.47	4,781.55
Q-UR	256	9.13%	2.56%	14,574.80	19,724.19
Q-LL	1823	65.09%	18.23%	1,394.04	694.24
Q-LR	633	22.60%	6.33%	4,399.14	929.46

DOX+ ADA 48h					
POPULATION	Count	% total	% Parent	Mean FL4-A	Mean FL3-A
ALL EVENTS	10,000	100%	100%	-	-
P1	3964	100%	39.64%	-	-
Q-UL	536	13.5	5.36%	1,601.42	5,413.17
Q-UR	1160	29.3	11.60%	7,699.55	21,977.25
Q-LL	1488	37.5	37.5%	1,433.50	983.61
Q-LR	780	19.7	7.80%	4,125.80	1,117.00

Q-UL	Quadrant-Upper Left
Q-UR	Quadrant-Upper Right
Q-LL	Quadrant-Lower LEFT
Q-LR	Quadrant-Lower Right

Table S4 and S5: Cell cycle distribution of MDA-MB-231 cells treated with DMSO, DOX, ADA, and the combination of DOX and ADA for 24 and 48hrs.

	24 Hour													
	Plot 1 (FSC-A/SSC-A)		Plot 6 (FL2-A)											
	All	P1	G0			G1			S			G2-M		
	Count	Count	Count	% of This Plot	Mean FL2-A	Count	% of This Plot	Mean FL2-A	Count	% of This Plot	Mean FL2-A	Count	% of This Plot	Mean FL2-A
B01 Control	10,000	6,797	152	2.23%	2,33,391.82	3,718	54.70%	14,12,518.32	2,501	36.80%	23,71,121.92	270	3.97%	38,00,871.87
B02 DOX	10,000	4,254	190	4.26%	2,42,985.69	723	16.23%	15,86,564.53	2,254	50.60%	25,74,681.30	917	20.60%	37,40,673.60
B05 ADA	10,000	6,487	247	3.80%	2,64,526.05	941	14.50%	15,99,538.47	4,140	63.82%	24,15,061.40	707	10.90%	38,17,023.37
B07 DOX ADA	10,000	2,313	298	12.90%	2,07,958.76	865	37.40%	15,36,817.27	948	41.00%	23,30,968.13	147	6.36%	37,98,249.89

	48 Hour													
	Plot 1 (FSC-A/SSC-A)		Plot 6 (FL3-A)											
	All	P1	G0			G1			S			G2-M		
	Count	Count	Count	% of This Plot	Mean FL3-A	Count	% of This Plot	Mean FL3-A	Count	% of This Plot	Mean FL3-A	Count	% of This Plot	Mean FL3-A
A03 48 CONTROL	10,000	5,336	207	3.86%	27,44,457.21	3,947	74.00%	52,52,193.27	1,279	23.97%	83,26,861.72	102	1.92%	1,22,31,779.96
A04 48 DOX	10,000	3,688	268	7.26%	17,03,786.80	546	14.80%	58,57,054.26	1,989	54.00%	92,61,948.25	819	22.20%	1,21,92,502.61
A07 48 ADA	10,000	2,389	214	8.96%	20,54,777.84	621	26.00%	58,15,266.12	1,402	58.70%	91,13,128.45	182	7.64%	1,21,93,350.10
A09 48 DOX ADA	10,000	2,967	288	9.39%	17,15,040.12	1118	37.70%	56,91,640.26	1,451	49.00%	87,09,863.14	183	6.16%	1,22,09,110.88
