# Supplementary material for: Adapalene and Doxorubicin Synergistically Promote Apoptosis of TNBC Cells by Hyperactivation of the ERK1/2 Pathway Through ROS Induction
Source: Front Oncol. 2022 Jul 6;12:938052. doi: 10.3389/fonc.2022.938052 (PMC9298514; doi:10.3389/fonc.2022.938052)
Supplement: Supplementary file 2 [file DataSheet_2.pdf]

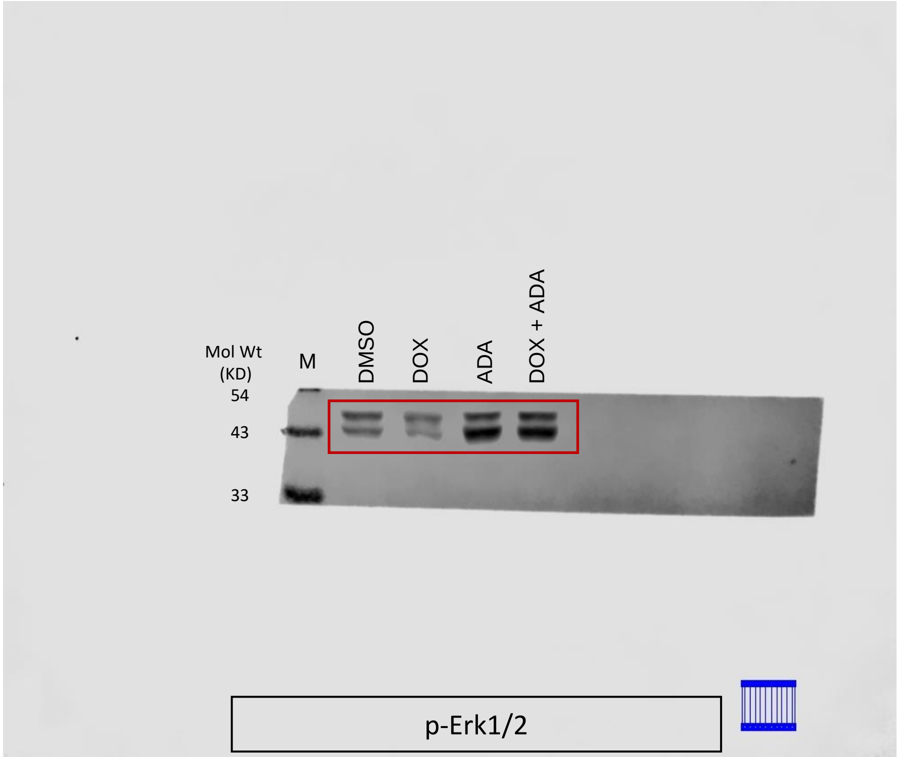

M- Marker (ladder)

DOX- Doxorbicin

ADA- Adapalene

DOX + ADA - Doxorubicin + Adapalene

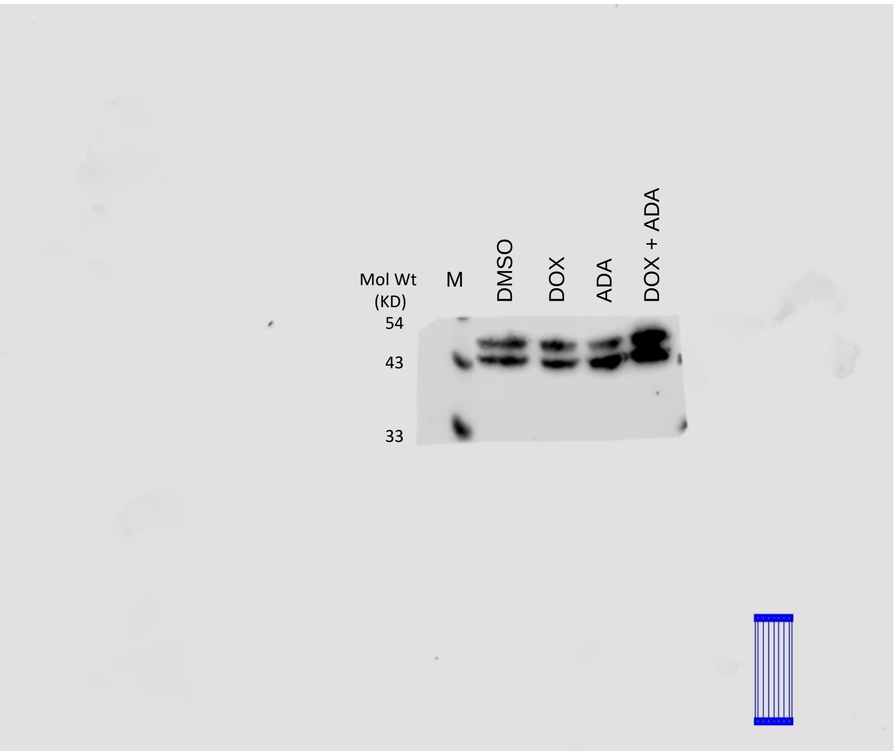

P-Erk1/2

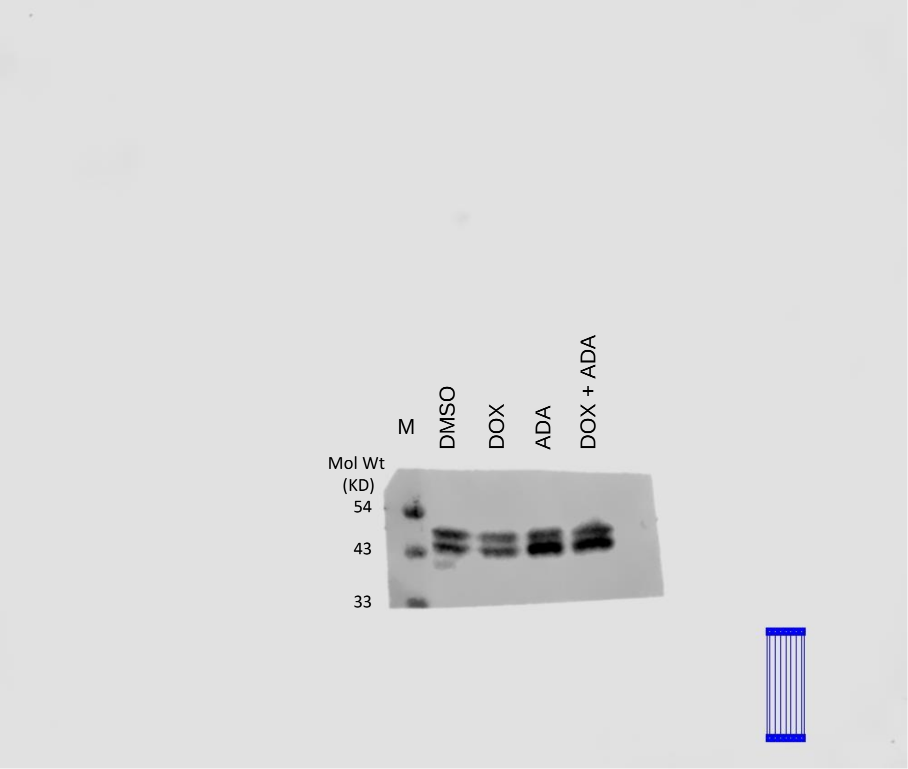
M
DMSO
DOX
ADA
DOX + ADA
Mol Wt
(KD)
54
43
33

P-Erk1/2

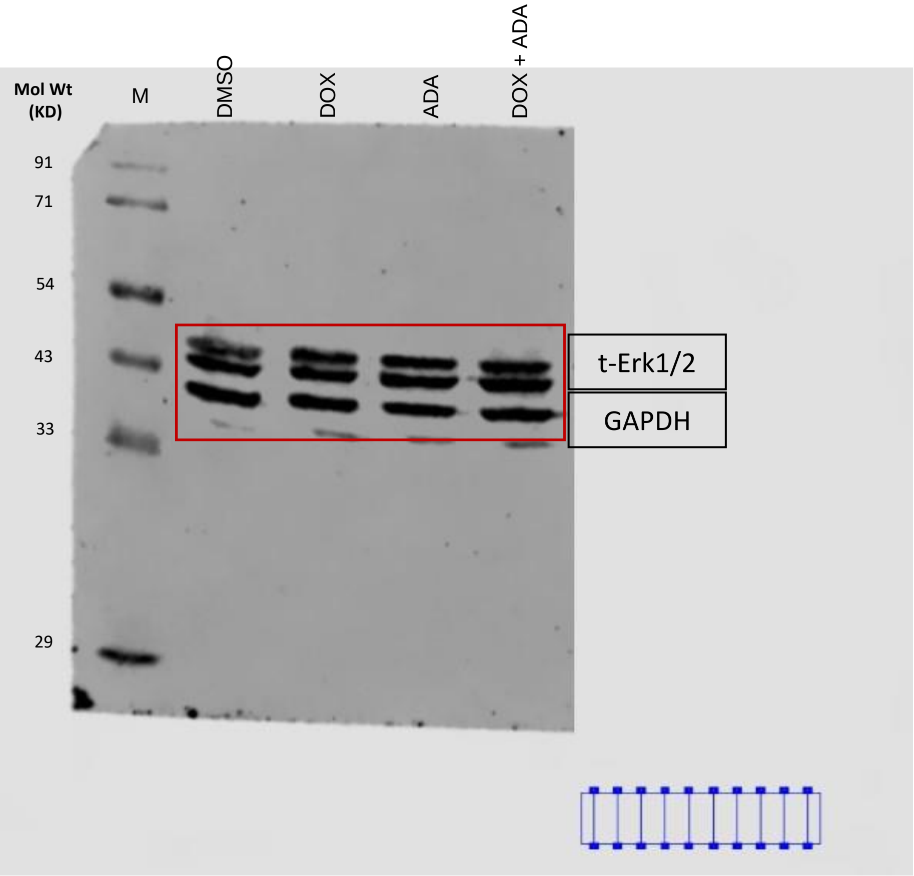
Mol Wt
(KD)
M
DMSO
DOX
ADA
DOX + ADA
91
71
54
43
33
29
t-Erk1/2
GAPDH

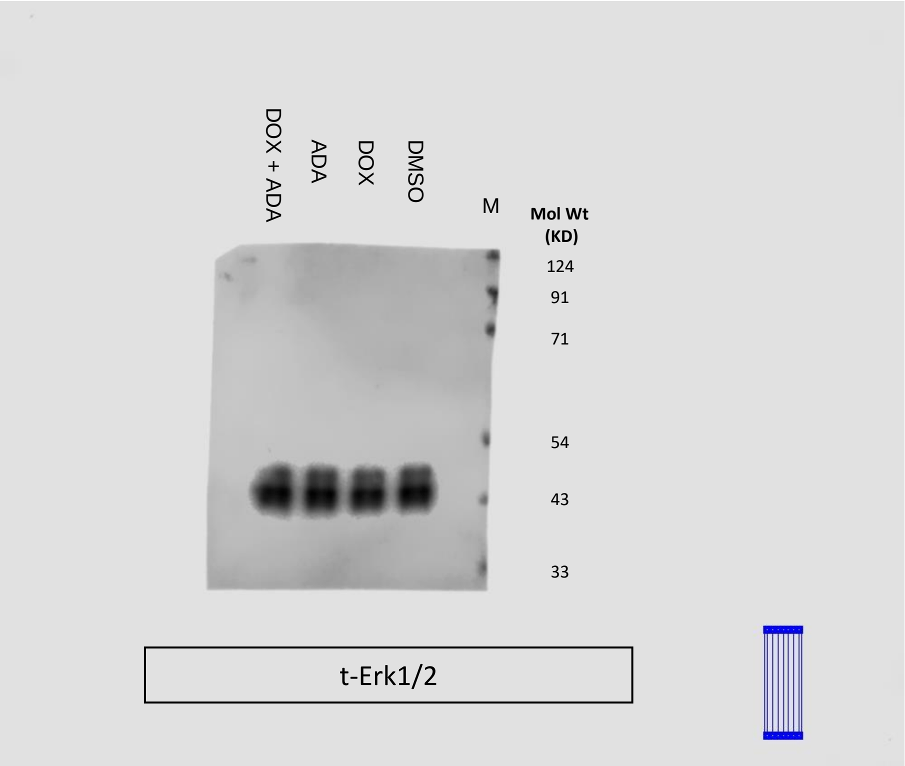
DOX + ADA
ADA
DOX
DMSO
M
Mol Wt
(KD)
124
91
71
54
43
33
t-Erk1/2

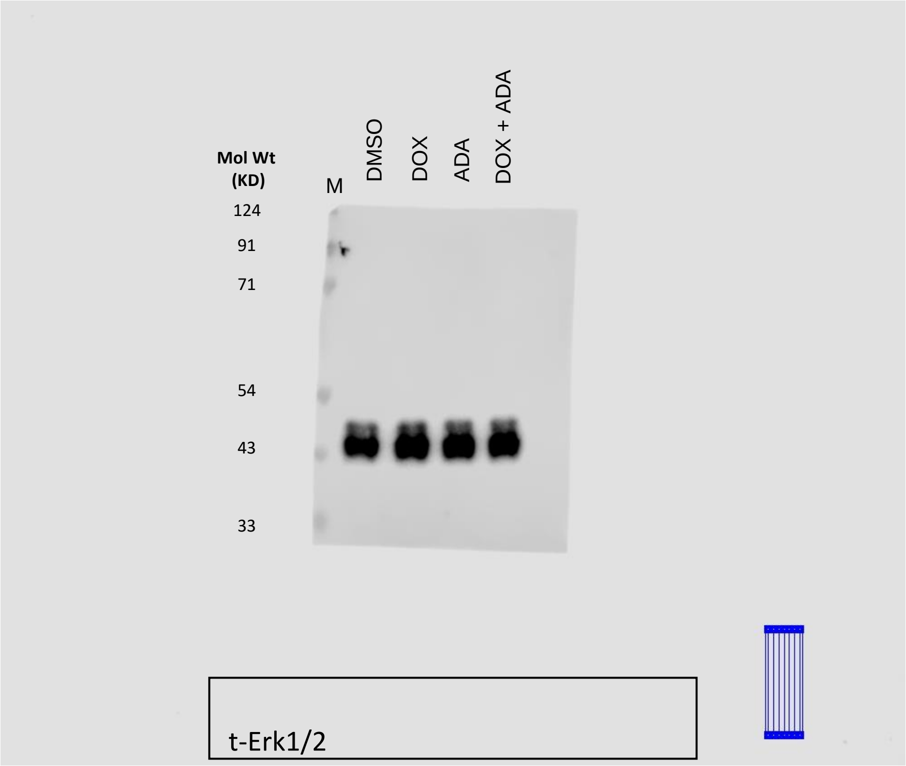
Mol Wt
(KD)
M
DMSO
DOX
ADA
DOX + ADA
124
91
71
54
43
33
t-Erk1/2

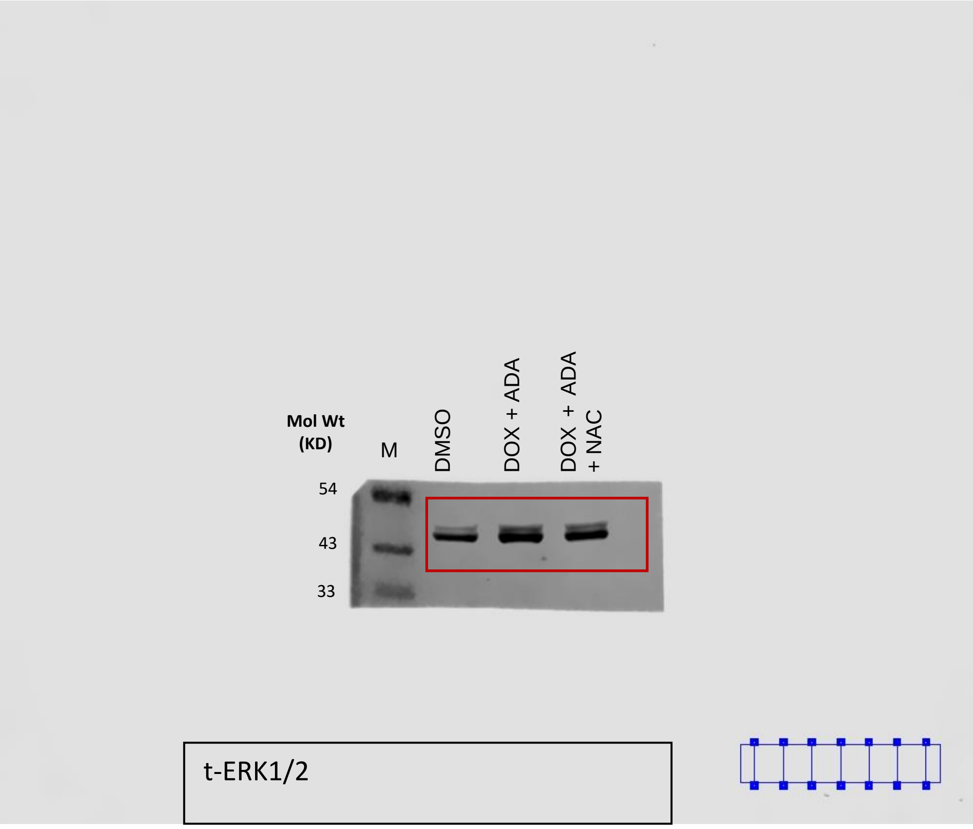
Mol Wt
(KD)
M
DMSO
DOX + ADA
DOX + ADA
+ NAC
54
43
33
t-ERK1/2

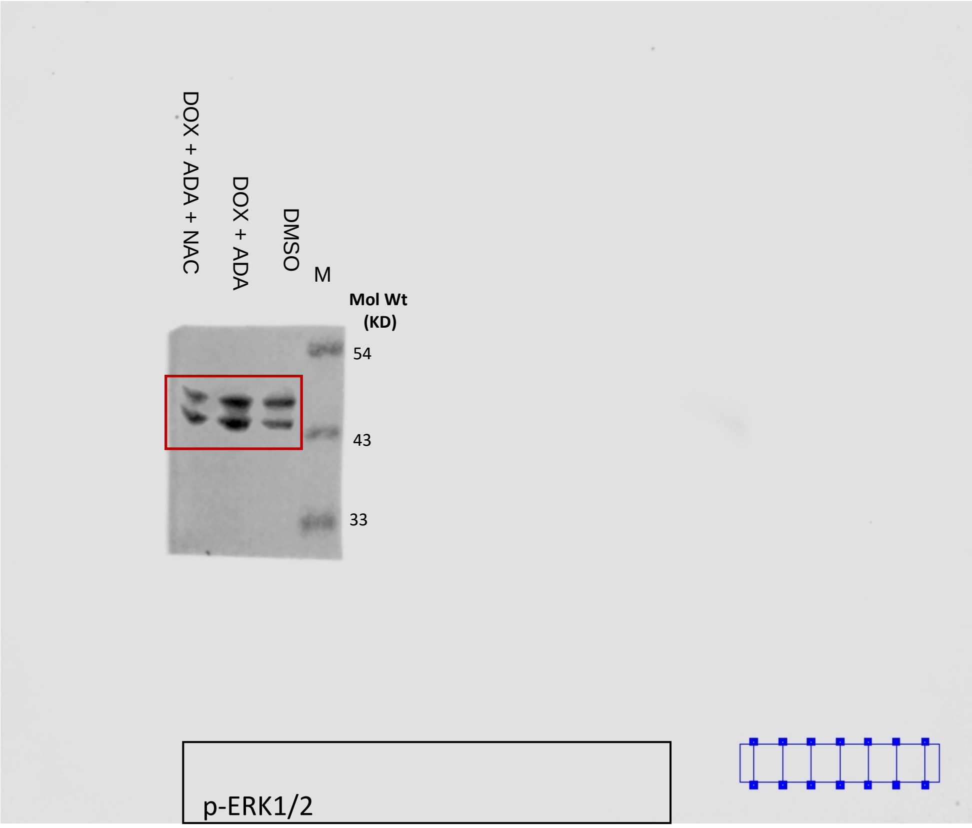
DOX + ADA + NAC
DOX + ADA
DMSO
M
Mol Wt
(KD)
54
43
33
p-ERK1/2

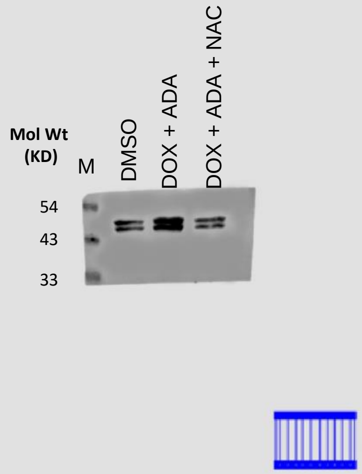

p-ERK1/2

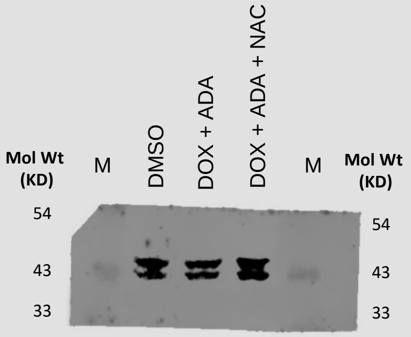

t-ERK1/2

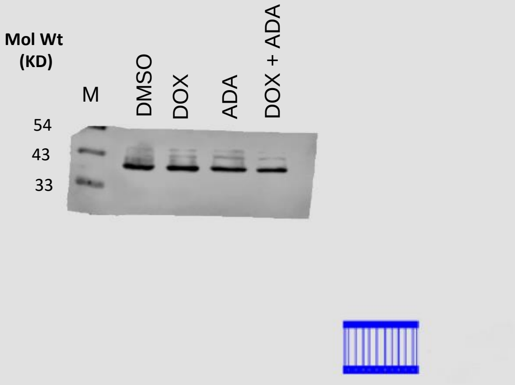
Mol Wt
(KD)
M
DMSO
DOX
ADA
DOX + ADA
54
43
33

GAPDH

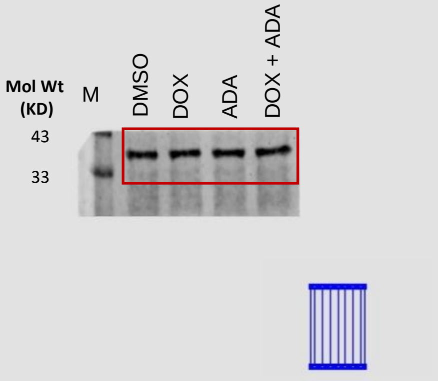
Mol Wt
(KD)
M
DMSO
DOX
ADA
DOX + ADA
43
33

GAPDH

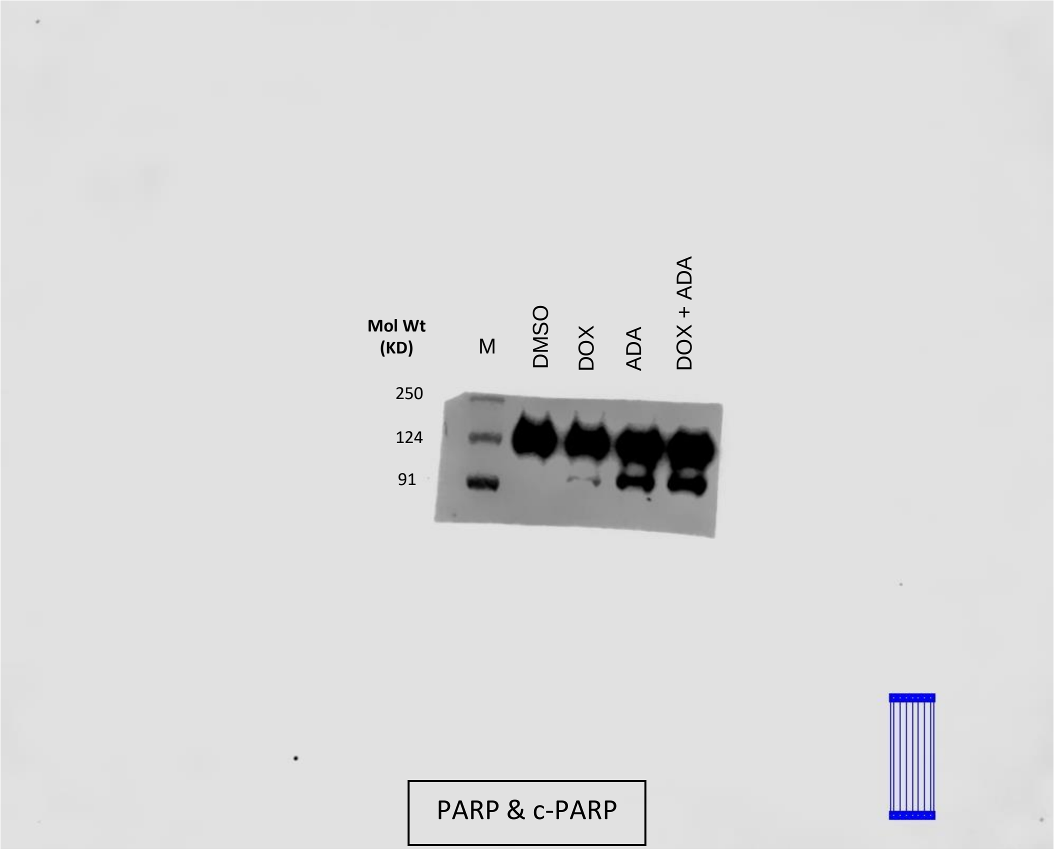
Mol Wt
(KD)
M
DMSO
DOX
ADA
DOX + ADA
250
124
91
PARP & c-PARP

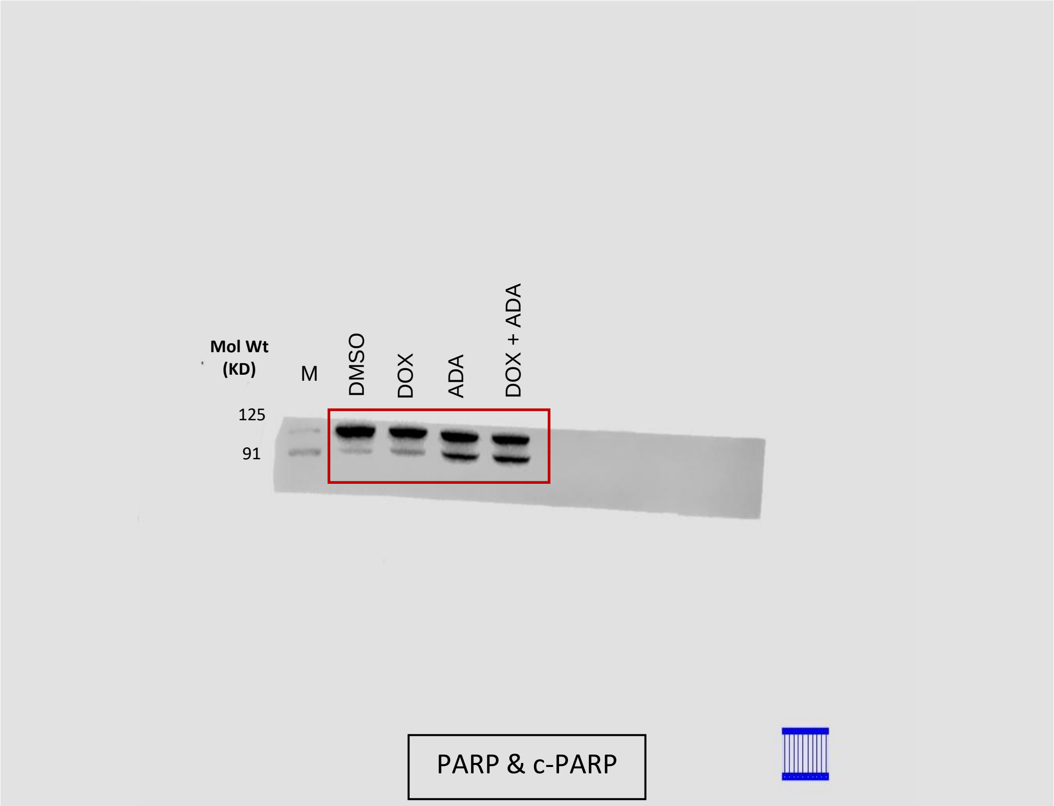
Mol Wt
(KD)
M
DMSO
DOX
ADA
DOX + ADA
125
91
PARP & c-PARP

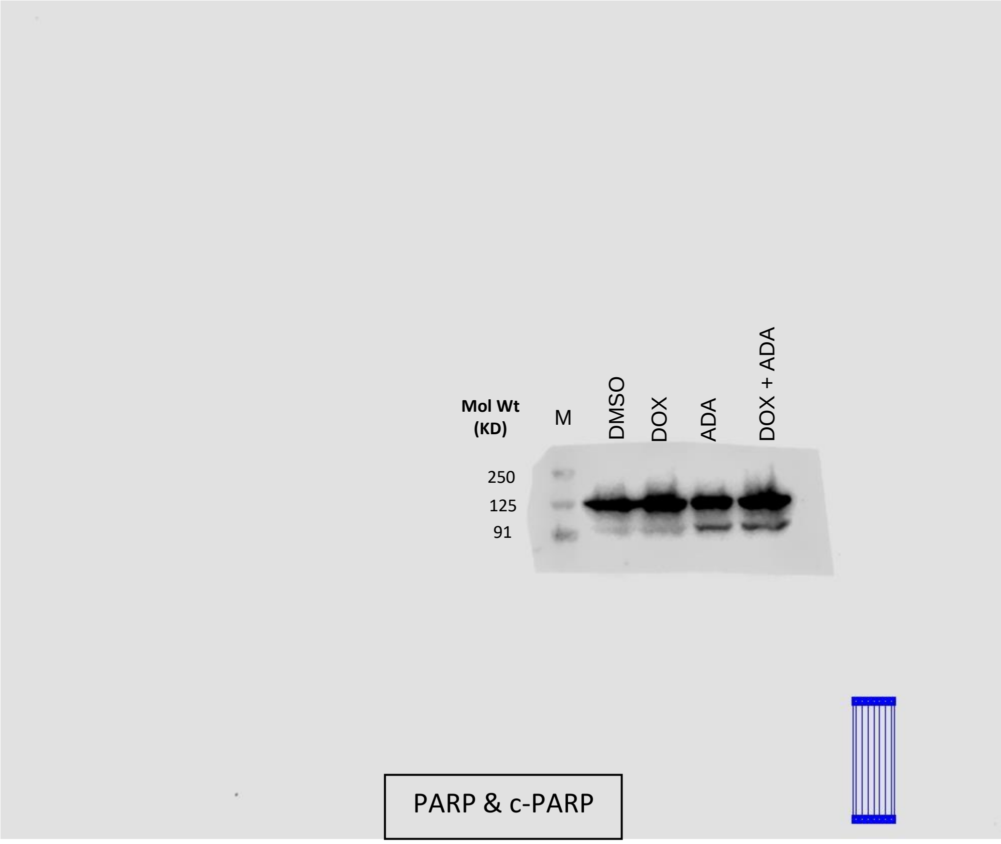
Mol Wt
(KD)
M
DMSO
DOX
ADA
DOX + ADA
250
125
91
PARP & c-PARP

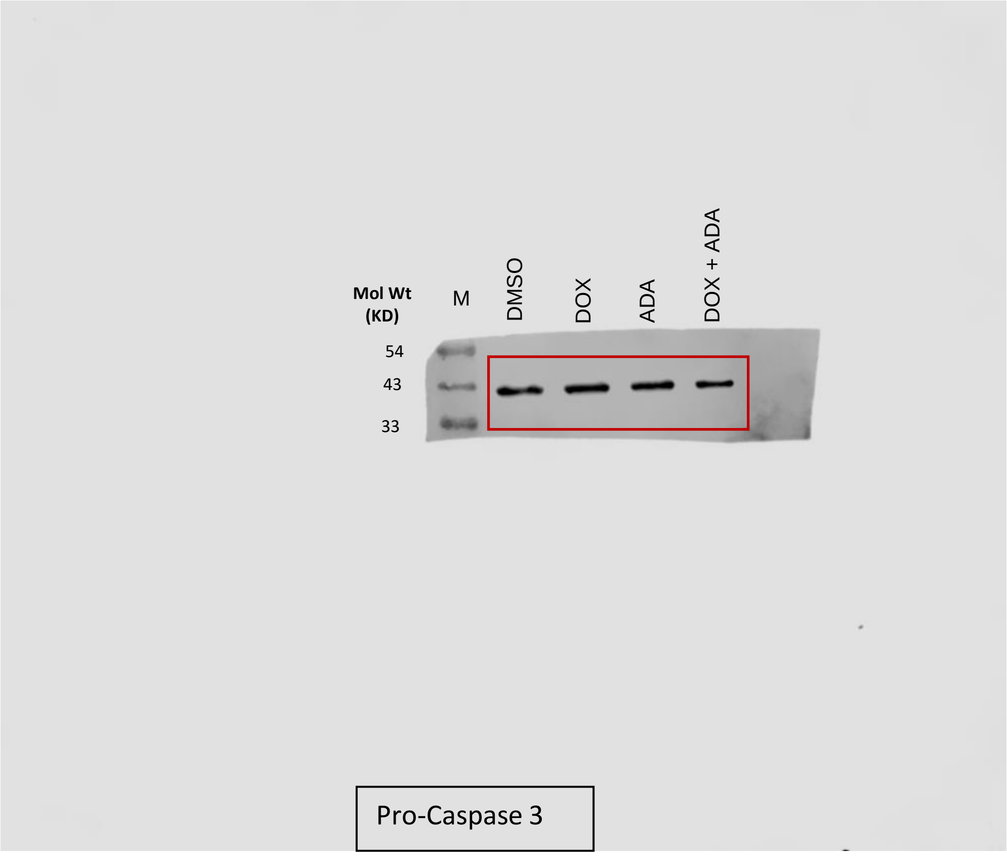
Mol Wt
(KD)
M
DMSO
DOX
ADA
DOX + ADA
54
43
33
Pro-Caspase 3

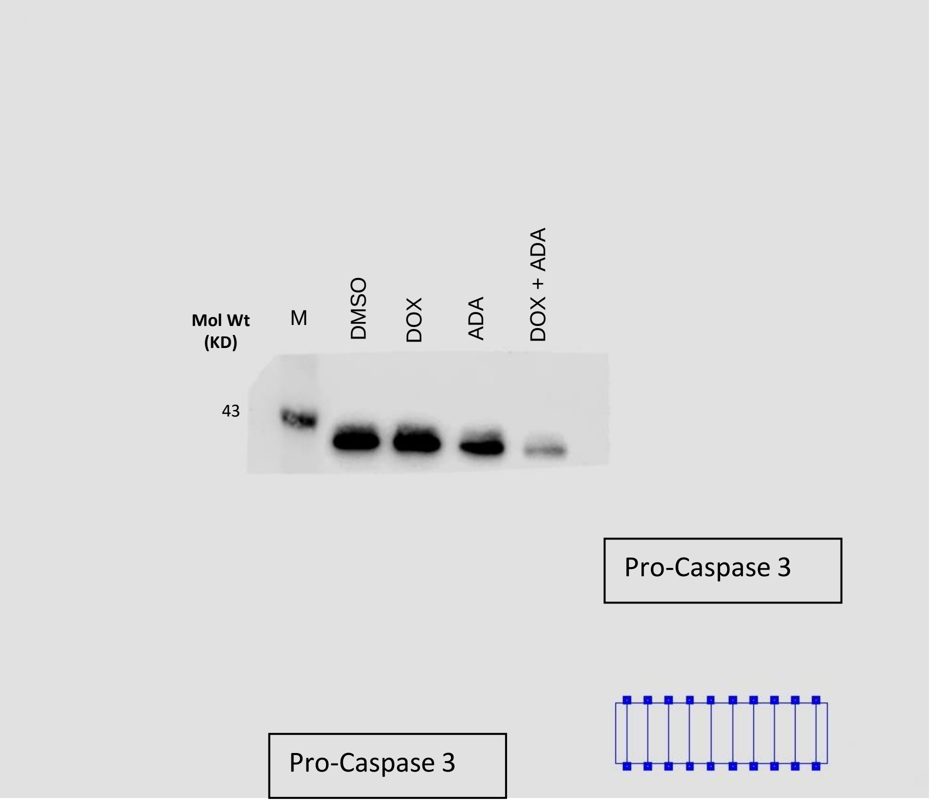
Mol Wt
(KD)
M
DMSO
DOX
ADA
DOX + ADA
43
Pro-Caspase 3
Pro-Caspase 3

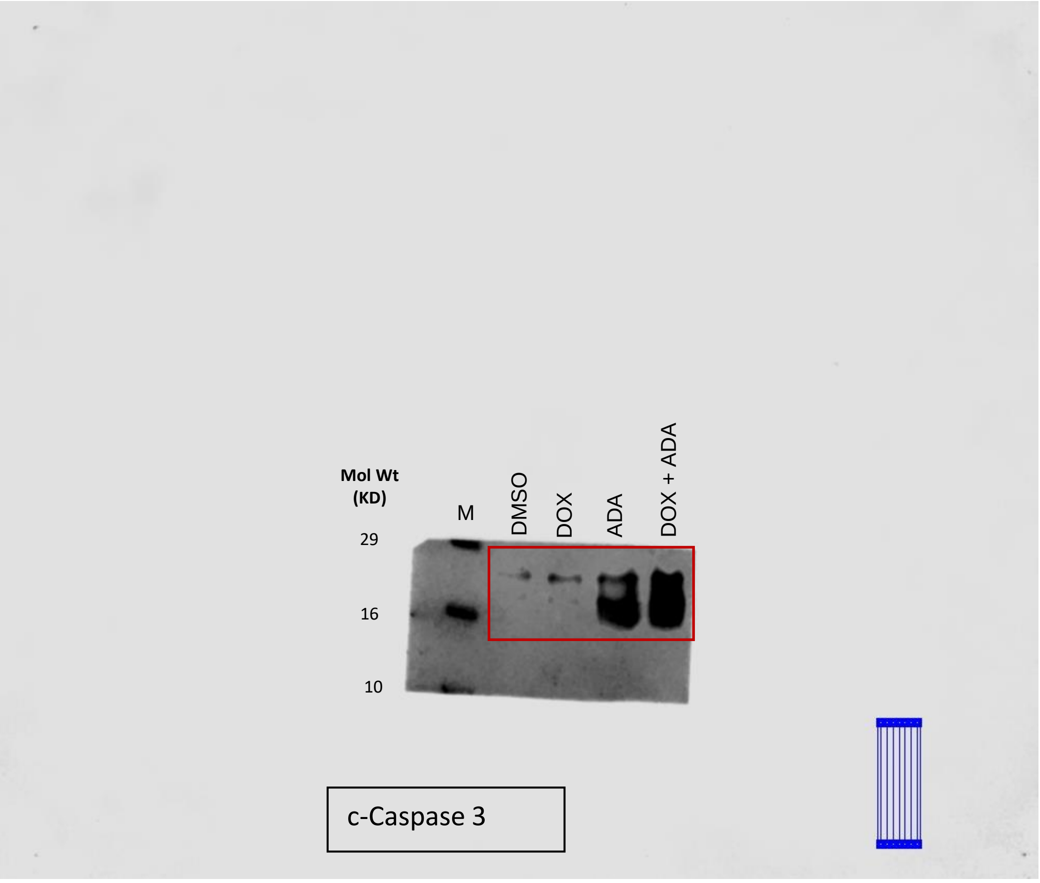
Mol Wt
(KD)
M
DMSO
DOX
ADA
DOX + ADA
29
16
10
c-Caspase 3

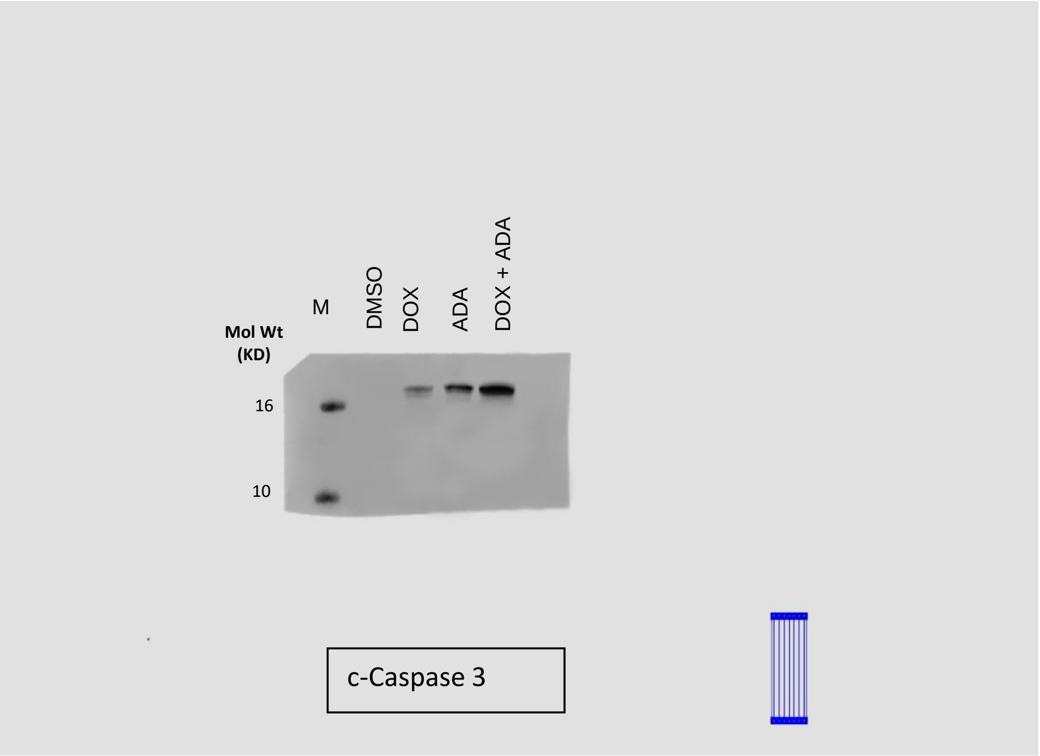
M
DMSO
DOX
ADA
DOX + ADA
Mol Wt
(KD)
16
10
c-Caspase 3

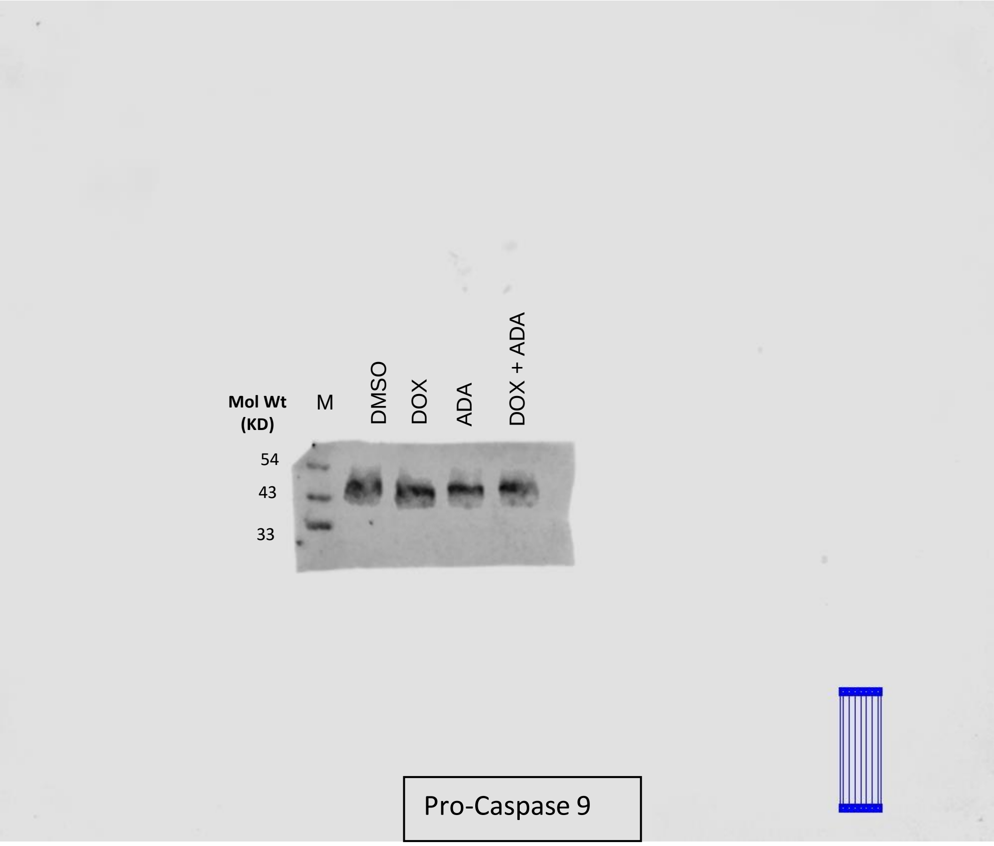

Mol Wt
(KD)
M
DMSO
DOX
ADA
DOX + ADA
54
43
33
Pro-Caspase 9

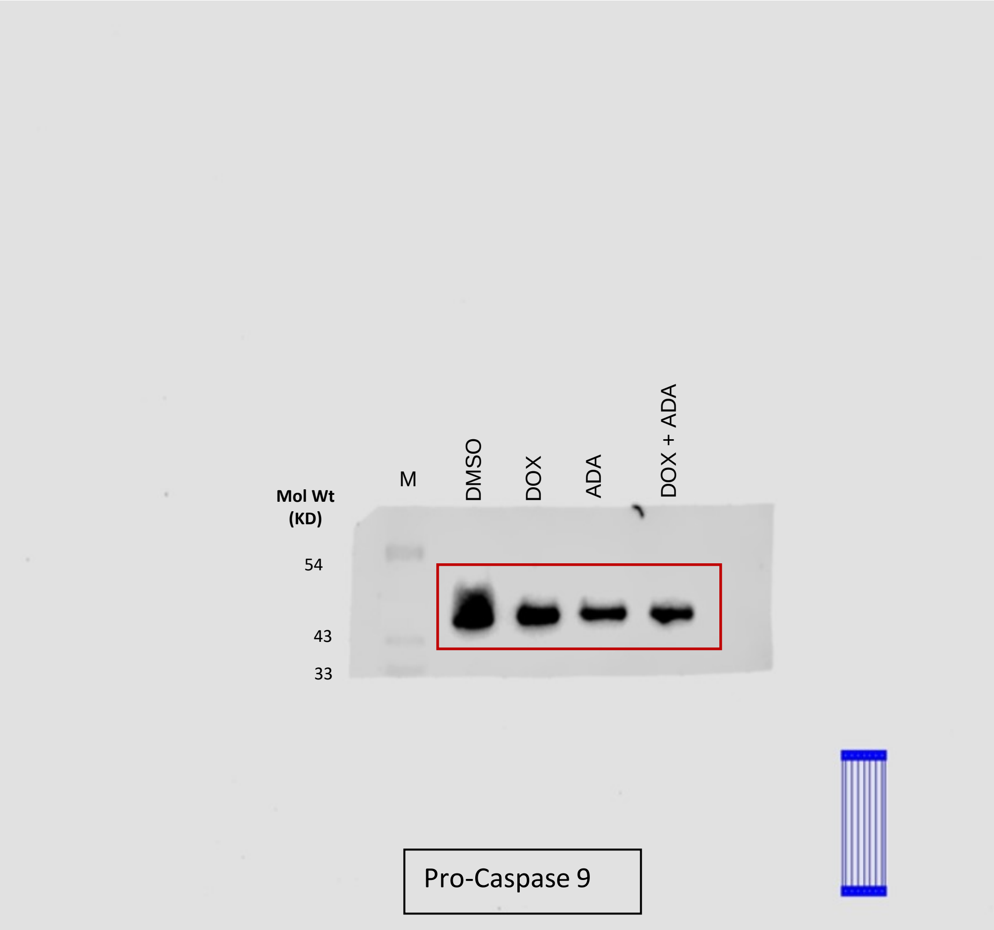

Mol Wt
(KD)
M
DMSO
DOX
ADA
DOX + ADA
54
43
33
Pro-Caspase 9

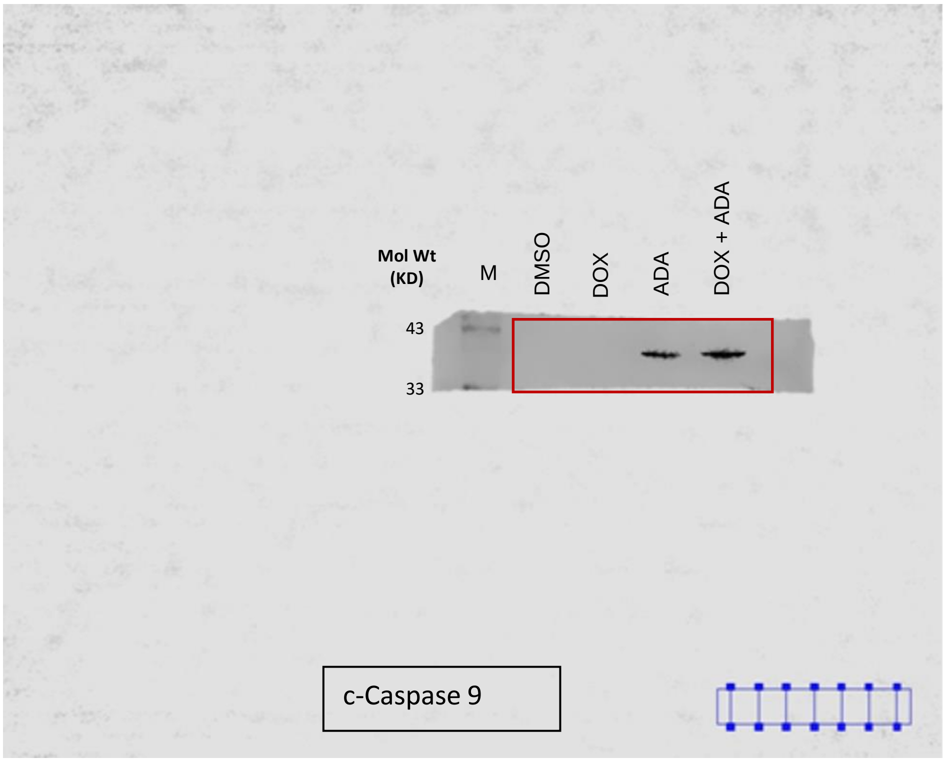
Mol Wt
(KD)
M
DMSO
DOX
ADA
DOX + ADA
43
33
c-Caspase 9

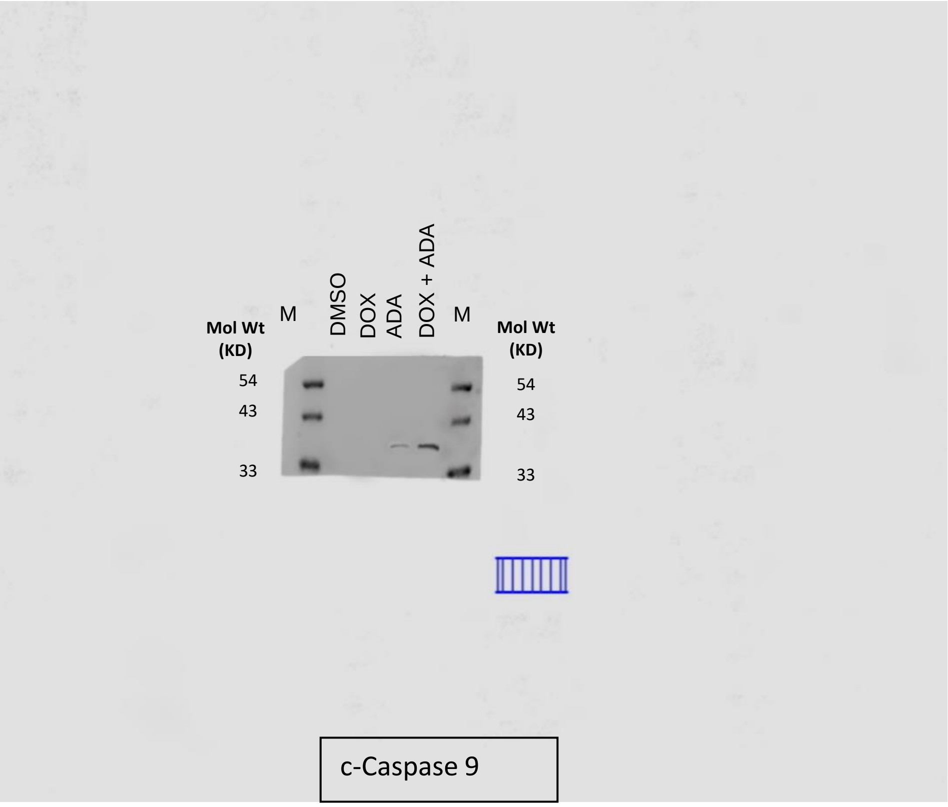
Mol Wt
(KD)
M
DMSO
DOX
ADA
DOX + ADA
M
Mol Wt
(KD)
54
43
33
54
43
33
c-Caspase 9
